# Supplementary material for: Unexpectedly uneven distribution of functional trade-offs explains cranial morphological diversity in carnivores
Source: Nat Commun. 2024 Apr 16;15:3275. doi: 10.1038/s41467-024-47620-x (PMC11021405; doi:10.1038/s41467-024-47620-x)
Supplement: Supplementary file 1 — Supplementary Information [file 41467_2024_47620_MOESM1_ESM.pdf]

Supplementary Information for "Unexpectedly uneven distribution of functional trade-offs explains cranial morphological diversity in carnivores"

Gabriele Sansalone[1,2,3†*], Stephen Wroe[2], Geoffrey Coates[2], Marie R. G. Attard[2,4], Carmelo Fruciano[1,5, 6†*]

[1] Institute for Marine Biological Resources and Biotechnology (CNR-IRBIM), National Research Council. Via S. Raineri – 4 98122 Messina, Italy

[2] Function, Evolution and Anatomy Research Lab, Zoology Division, School of Environmental and Rural Science, University of New England, Armidale, NSW, Australia

[3] University of Modena and Reggio Emilia, Department of Life Sciences, Via Campi 213D, 41125, Modena, Italy

[4] British Antarctic Survey, Natural Environment Research Council, High Cross, Madingley Road, Cambridge CB3 0ET, UK

[5] National Biodiversity Future Center, Palermo, Italy

[6] Department of Biological, Geological and Environmental Sciences, University of Catania, via Androne 81 – 95124 Catania, Italy

***Corresponding authors:** Gabriele Sansalone, Carmelo Fruciano Email: gabriele.sansalone@unimore.it; carmelo.fruciano@unict.it

[†] Current addresses: University of Modena and Reggio Emilia, Department of Life Sciences, Via Campi 213D, 41125, Modena, Italy; Department of Biological, Geological and Environmental Sciences, University of Catania, via Androne 81 – 95124 Catania, Italy

Supplementary Methods

Supplementary Table 1. Root mean square error (RMSE) values for each interpolating approach and for each performance metrics.

**Polynomial degree**	**Bite force**	**Bite velocity**
1st	0.192	0.146
2nd	0.143	0.117
3rd	0.132	0.085
4th	0.104	0.068
5th	0.107	0.074
**TPS degree**		
2nd	**0.042**	0.019
3rd	0.047	**0.018**
4th	0.053	0.023
**Kriging model**		
Spherical	0.216	0.203
Exponential	0.059	0.031
Gaussian	0.059	0.296

Evolutionary modelling

We used EIC to calculate the relative support for each evolutionary model. The models' EIC display considerable overlap (Supplementary Figure 1) suggesting that BM, EB and OU models have similar fit. We assessed the mode of evolution of the weight *w* by fitting different models using the function fitContinuous accounting for standard error and the results are reported in Supplementary Table 4.

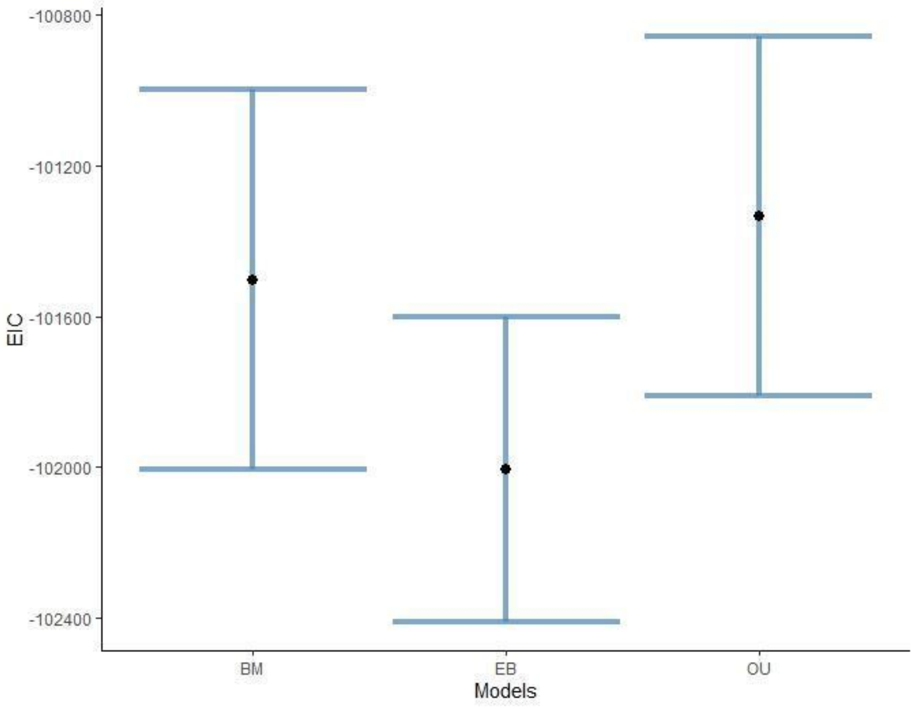

Supplementary Figure 1. Point estimate +/- 2 standard error of EIC for different evolutionary models fitted on cranial shape. Source Data are available at https://doi.org/10.6084/m9.figshare.23553648 in the “Code and Source Data” folder, included in the Supp_Fig1.rda file.

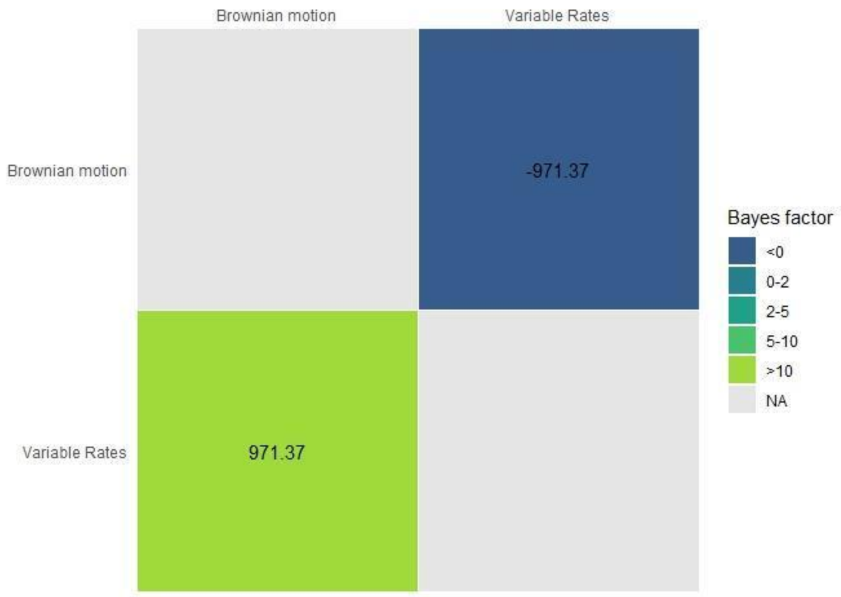

Supplementary Figure 2. Bayes factors computed for variable rates and single rate (Brownian motion) models fitted on cranial shape. Large positive values at one cell suggest support for the model in the corresponding row compared to the model in the corresponding column.

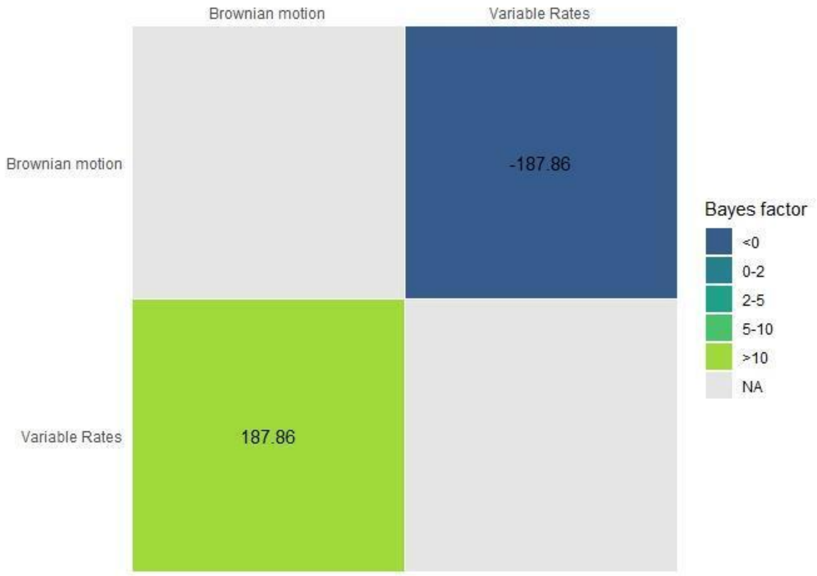

Supplementary Figure 3. Bayes factors computed for variable rates and single rate (Brownian motion) models fitted on weight *w*.

Shape analysis

We digitised 35 homologous landmarks on 49 of the total 132 species included in this study. The landmarks were digitised on 3D surfaces using the IDAV landmark software (see Supplementary Table 1 for details on landmark definitions). The remaining species were derived from the dataset published by Law[1], as our landmarks configuration represents a subset of the landmarks used by this source. To ensure that the two datasets could be combined, we first aligned the dataset from Law[1] to the space defined by our 49 species using ordinary Procrustes analysis as implemented in the function procOPA from the R package shapes[2]. Where possible, we included multiple specimens per species. To account for the potential effect of sexual dimorphism on the shape data,

we performed a repeated measure test[3] considering males and females as repeated measures of the same species without finding significant differences ($F = 0.22$; $P = 0.87$). Then, we extracted the species shared between the two dataset and performed a repeated measure test, which failed to reject the null hypothesis of no difference in shape between corresponding shapes between the two datasets ($F = 2.23$; $P = 0.15$). This suggests that the species-level shape estimates are not consistently different between datasets. Furthermore, we tested whether potential differences between the two datasets could arise from size differences in the specimens used. To do so we computed the Procrustes distances between corresponding species in the two datasets and the absolute differences between the centroid sizes of the different configurations. Then we tested for the significance of the correlation of these Procrustes distances and differences in centroid size and found they were not significant (Pearson $r = 0.31$, $P = 0.073$; Spearman *rho* = 0.29, $P = 0.084$). These two analyses – and particularly the lack of significance in the repeated measures test on shape – provided us with the necessary confidence to combine the two datasets as we do not find consistent differences (bias) in shape between datasets so combining them should not negatively affect downstream analyses.

We further evaluated digitization error in our dataset by generating three replicates of our dataset (49 species). The three replicates were digitized in three consecutive days by the same operator (GS). Then we compared, separately, each of three replicates and the average of the replicates with the original digitization using a repeated measure test[3]. In each case we did not find a significant difference between the original and replicate digitisations ($F = 0.33$; $P = 0.57$ and $F = 0.096$; $P =$ 0.90 respectively).

Supplementary Table 2. Position of landmarks used in this study.

Landmark	Position
1	Anteriormost point of premaxilla
2-3	Right-left anteriormost point of canine alveolus
4-5	Anteriormost point of premolar alveolus
6-7	Anteriormost point of first molar alveolus
8-9	Posteriormost point of last molar alveolus
10-11	Ventralmost point of pterygoid hamulus
12-13	Medialmost point of mandibular fossa
14-15	Ventralmost point on mastoid process
16-17	Dorsalmost point of external edge of auditory meatus
18-19	Ventralmost point of external edge of auditory meatus
20-21	Lateralmost point on occipital condyle
22-23	Dorso-ventral borders of foramen magnum
24	Posteriormost point of midline palate
25	Anteriormost point of palate
26	Anteriormost point of midline of nasals
27	Posterior intersection of lamboid and sagittal crests
28-29	Anteriormost point of the orbit
30-31	Posteriormost point of the intersection between zygomatic arch and the braincase
32-33	Ventralmost point of intersection between the zygomatic arch and the maxilla
34-35	Lateralmost point of the mandibular fossa

Replication of macroevolutionary analyses using direct bite force estimates

We repeated the main analyses presented in this study using the bite forces estimated from our Finite element models, as detailed in the following steps:

1) The bite force values for the species in our study were compared to those obtained from previous studies[4–7]. Bite force estimates in previous studies – and their variation among species - are largely consistent, supporting the robustness of our simulations.

2) We computed the correlation between von Mises stress values and bite force values estimated from our models. Results indicated a strong correlation between the two metrics (Pearson $r = 0.82$, p-value < 0.001; Spearman $rho = 0.88$; p-value < 0.001).

3) We estimated the best fitting interpolation strategy following the framework described in the main text, and a second degree TPS showed the lowest RMSE for bite force values. The performance surface for bite force directly estimated form the models (Supplementary Figure 5) is, indeed, very similar to the one displayed in Figure 1c based on von Mises stress

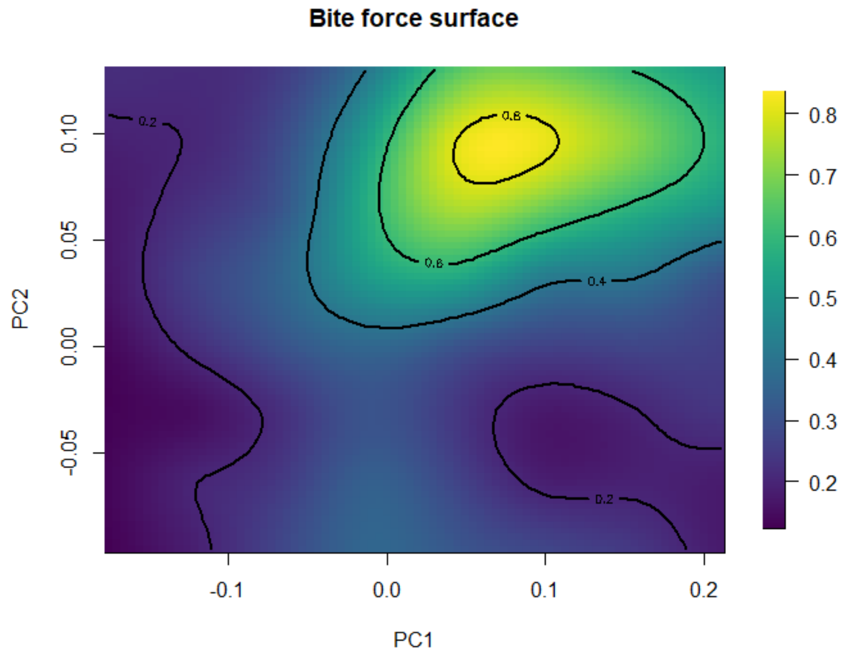

Supplementary Figure 4. Performance surface estimated for direct estimates of bite force from Finite element models. Source Data are available at https://doi.org/10.6084/m9.figshare.23553648 in the “Code and Source Data” folder, included in the Supp_Fig4.rda file.

4) We computed the new trade-off weight *w* values based on bite force values and used them in downstream analyses.

5) We computed evolutionary rates for bite force values using the same approaches (Bayesian estimates from BayesTraits and Ridge Regression from RRphylo) described in the Methods section. We computed correlations between shape evolutionary rates and trade-off weight *w* values (based on bite force values) evolutionary rates. Results showed that shape rates were again uncorrelated with the weight *w* rates based on bite force values (Pearson *r* = 0.026, p-value = 0.24; Spearman *rho* = 0.013, *p*-value = 0.81). The same holds when using phylogenetically corrected tip rates values (Pearson *r* = 0.022, *p*-value = 0.81; Spearman *rho* = 0.12, *p*-value = 0.16). The distribution of rates of the trade-off weight *w* based on bite force values (Supplementary Figure 6) is highly comparable with the distribution of rates of weight w based on von Mises stress as displayed in Fig. 2b-d.

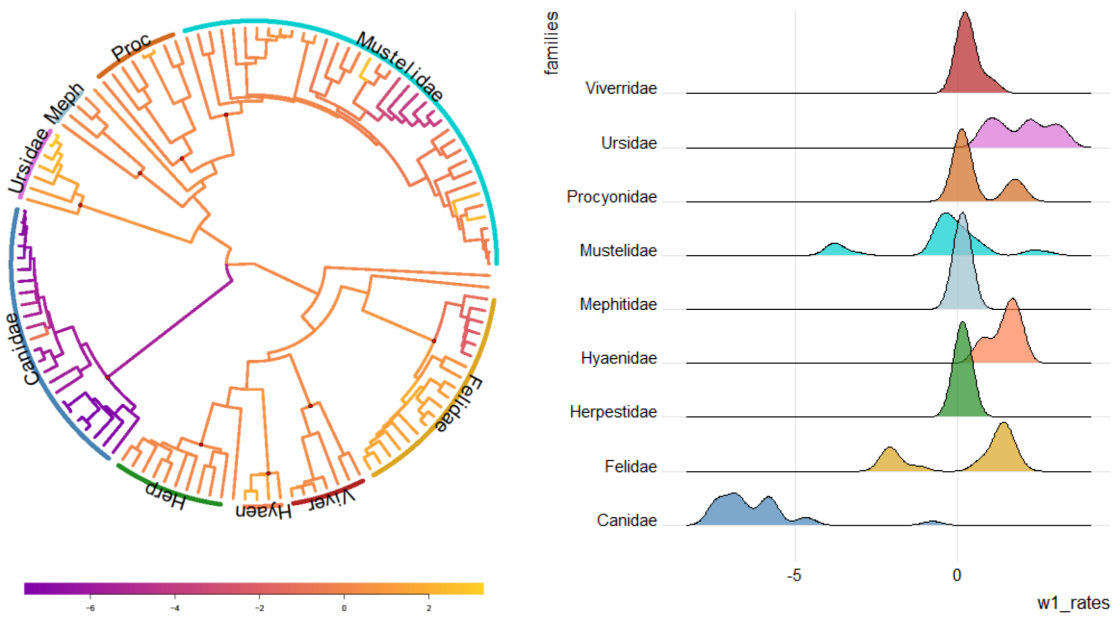

Supplementary Figure 5. Distribution of trade-off weight *w* rates of evolution on the carnivore phylogeny (left panel). Density ridge plot of per clade trade-off weight *w* rates (right panel). Source Data are available at https://doi.org/10.6084/m9.figshare.23553648 in the “Code and Source Data” folder, included in the Supp_Fig5.rda file.

We then computed correlations between trade-off weight *w* tip rates based on von Mises stress and trade-off weight *w* tip rates based on bite force values. Results showed that tip rates of both trade-off weights *w* were significantly correlated (Pearson *r* = 0.71, *p*-value < 0.001;Spearman *rho* = 0.83,

p-value < 0.83). The same holds when using phylogenetically corrected tip rates values (Pearson r = 0.67, *p*-value < 0.001; Spearman *rho* = 0.72, *p*-value < 0.73).

6) We repeated our analysis to understand the relationship between the force-velocity trade-off and morphological disparity. Again, we used the same approach described in the Methods section, but using the trade-off weight w based on estimated bite force values. The distributions of weight w volume and morphological disparity (measured as multivariate variance) showed an overlapping pattern, with three identifiable peaks (see Supplementary Figure 7). However, there are some differences in the shape of the peaks when compared to the distribution obtained using the weight w based on von Mises stress data. In particular, the peak around values of 0.41 which was higher. Nonetheless, morphological disparity and weight w volume were positively correlated (Pearson r = 0.77, p-value < 0.001; Spearman *rho* = 0.58, *p*-value < 0.001; *Xi* = 0.63, *p*-value < 0.001).

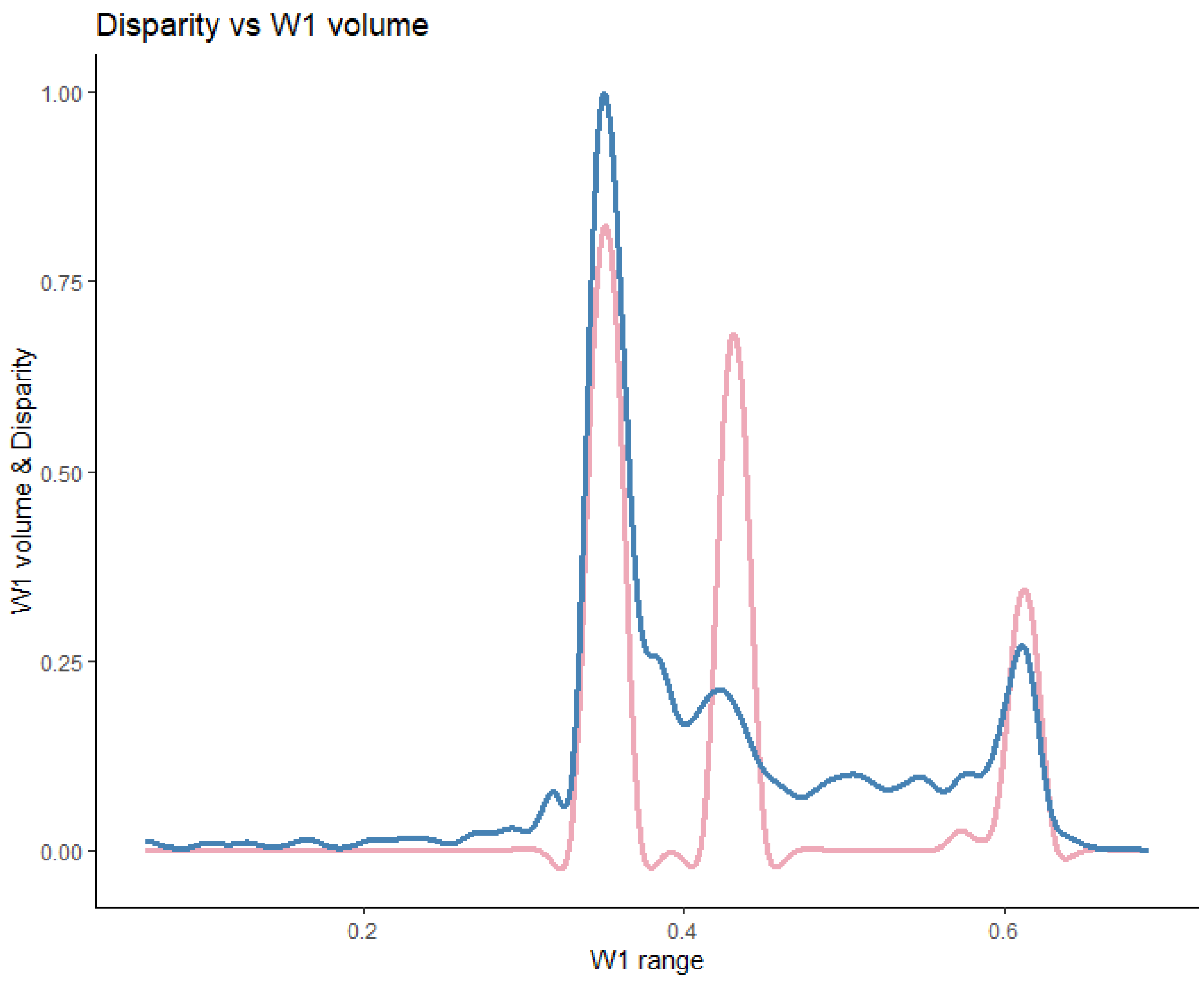

Supplementary Figure 6. **Sliding-window-based distribution of actual and theoretical morphological disparity at varying levels of trade-off weight *w* (estimated using bite force values).** The pink line indicates actual morphological disparity at a window corresponding to a given weight *w*, the blue line indicates the weight *w* volume. The values have been scaled to range between zero and one to be comparable. Source Data are available at https://doi.org/10.6084/m9.figshare.23553648 in the "Code and Source Data" folder, included in the Supp_Fig6.rda file.

Finite elements analysis (FEA)

The volume meshes for the specimens in our sample used in FEA (Supplementary Data 3) were then assigned 8 different material properties, intended to represent the actual material properties of mammalian bone[8] and are reported in Supplementary Table 3. Furthermore, we checked for the potential discrepancies in mesh homogeneity by computing the two indices[9]; the PEofAM (Percentage Error of the Arithmetic Mean) and PEofM (Percentage Error of the Median) percentages for our 49 real models and for the 64 theoretical geometries. Overall, our meshes are sufficiently homogeneous as they ranged between 0.21% and 2.36% (median = 0.83%) for PEofAM and between 1.78% and 5.22% (median = 2.53%) of PEofM, suggesting that raw stress data could be safely used for further statistical analyses. In our analyses we used the inverse of von Mises stress rather than the reaction forces at the biting point as VM stress data are collected over the entire geometry of the skull. Muscle forces have been estimated using Thomason's dry skull method[10], which can predict muscle forces also in extinct species. Previous biases reported are very low and statistically insignificant[11]. Recent studies confirm the dry skull method to be a highly supported strategy and is particularly useful for fossil taxa[12].

Supplementary Table 3. The values assigned to each material property in the finite element models, along with the colour designated to each property. The colours operate on a greyscale, with less dense materials allocated darker colours and higher density materials allocated lighter colours (see [8] for details).

Material Property Number	Modulus Value	Poisson Ratio	Designated Colour
1	1529.72	0.4	
2	1868.60	0.4	
3	2223.60	0.4	
4	10786.84	0.4	
5	21734.22	0.4	
6	27082.16	0.4	
7	32704.30	0.4	
8	38575.44	0.4	

Supplementary Table 4. AICc values of different evolutionary models fitted for trade-off weight *w*.

	BM	EB	OU
AICc	**-324.87**	-322.73	-314.18

Evolutionary rates

We evaluated the correlation between results generated from BayesTraitsV4.0 and RRphylo using Pearson correlation coefficients. Furthermore, we evaluated correlation between tip rates using the *Xi* coefficient[13] which is particularly suited for non-linear and non-monotonic relationships. To

account for shared ancestry, we repeated the above tests after computing phylogenetic independent contrasts (PIC)[14] of tip rates estimated by Bayes Traits and RRphylo. Results are reported in Supplementary Table 5. Importantly, these significant correlations strengthen our confidence in the patterns of variation in rates we document and support using one of the two methods (BayesTraits) in the main text and downstream analyses. These results also suggest that using the first 20 phylogenetic PCs for the analysis of shape in BayesTraits did not affect the results (the method in RRphylo uses all shape information).

Supplementary Table 5. Correlation tests between evolutionary rates estimated by BayesTraitsV4.0 and RRphylo. Statistical tests are one-sided.

	**Pearson r**	**Xi**	**PIC Pearson r**	**PIC Xi**
Cranial shape rates	0.71<br>$P < 0.001$	0.54<br>$P < 0.001$	0.59<br>$P < 0.001$	0.36<br>$P <0.001$
Weight *w* rates	0.82<br>$P < 0.001$	0.38<br>$P < 0.001$	0.64<br>$P < 0.001$	0.35<br>$P <0.001$

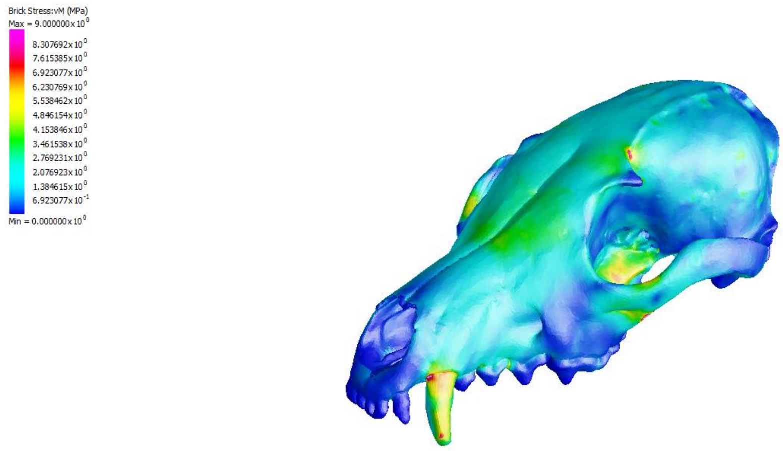

Supplementary Figure 7: Finite element model of a red fox (*Vulpes vulpes*) cranium. The models in Supplementary Figures 7 to 55 have been scaled to the same colour range. Models are not to scale.

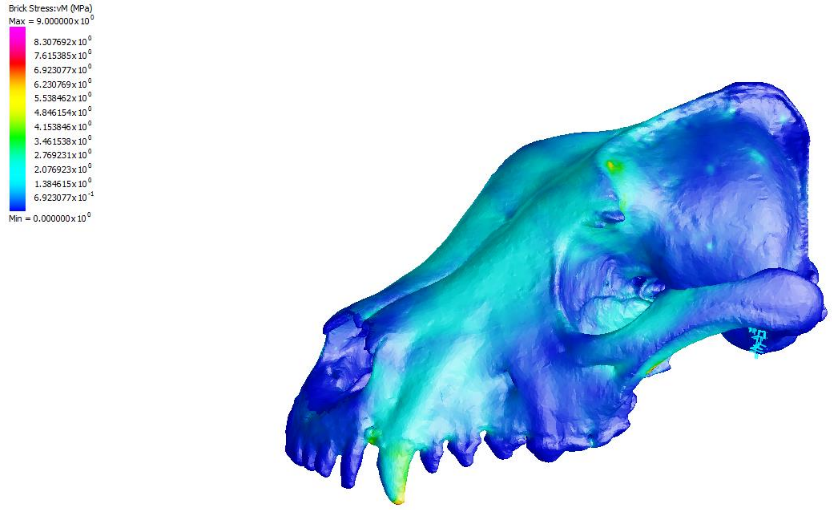

Supplementary Figure 8: Finite element model of a wild dog (*Lycaon pictus*) cranium. The models in Supplementary Figures 7 to 55 have been scaled to the same colour range. Models are not to scale.

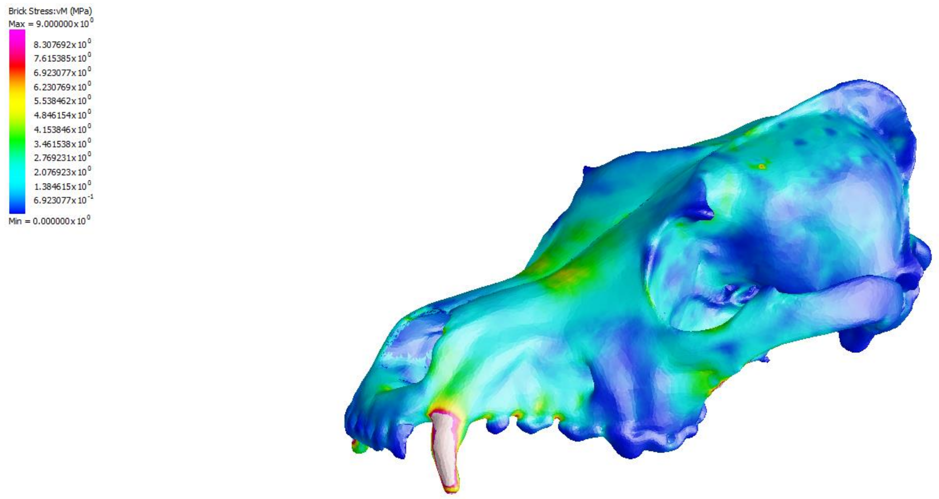

Supplementary Figure 9: Finite element model of a dingo (*Canis lupus dingo*) cranium. The models in Supplementary Figures 7 to 55 have been scaled to the same colour range. Models are not to scale.

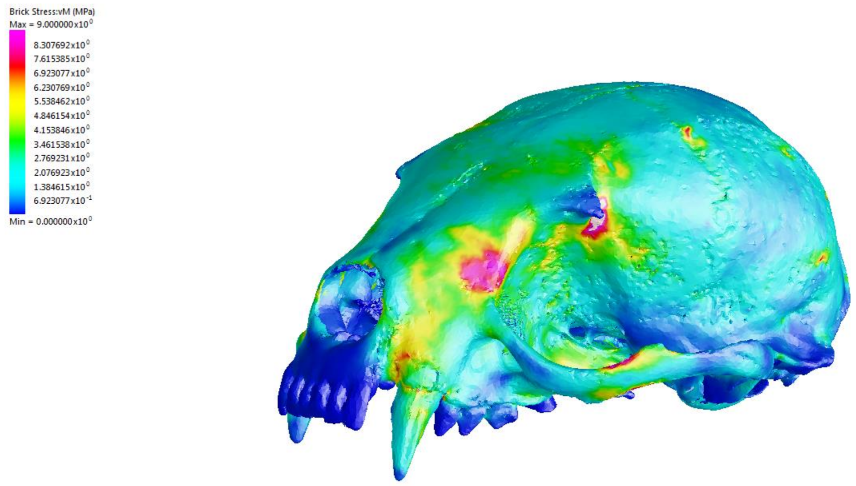

Supplementary Figure 10: Finite element model of a kinkajou (*Potos flavus*) cranium. The models in Supplementary Figures 7 to 55 have been scaled to the same colour range. Models are not to scale.

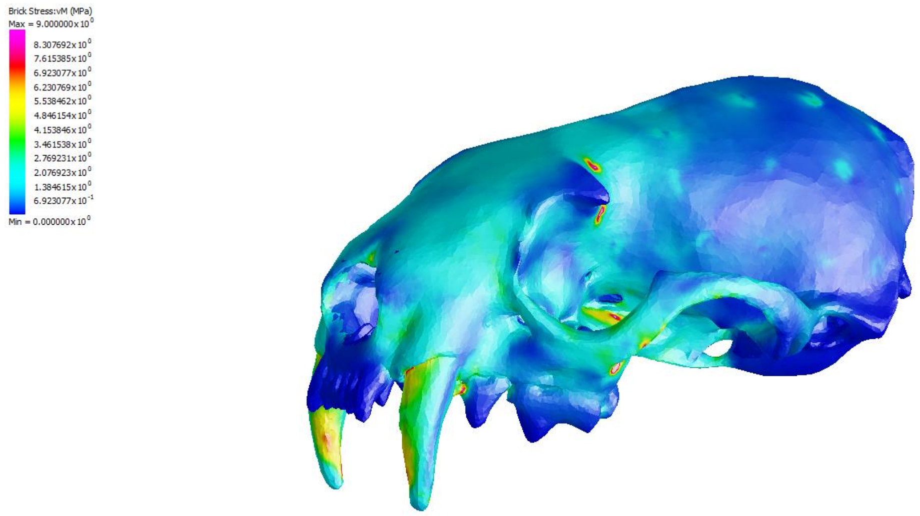

Supplementary Figure 11: Finite element model of a European polecat (*Mustela putorius*) cranium. The models in Supplementary Figures 7 to 55 have been scaled to the same colour range. Models are not to scale.

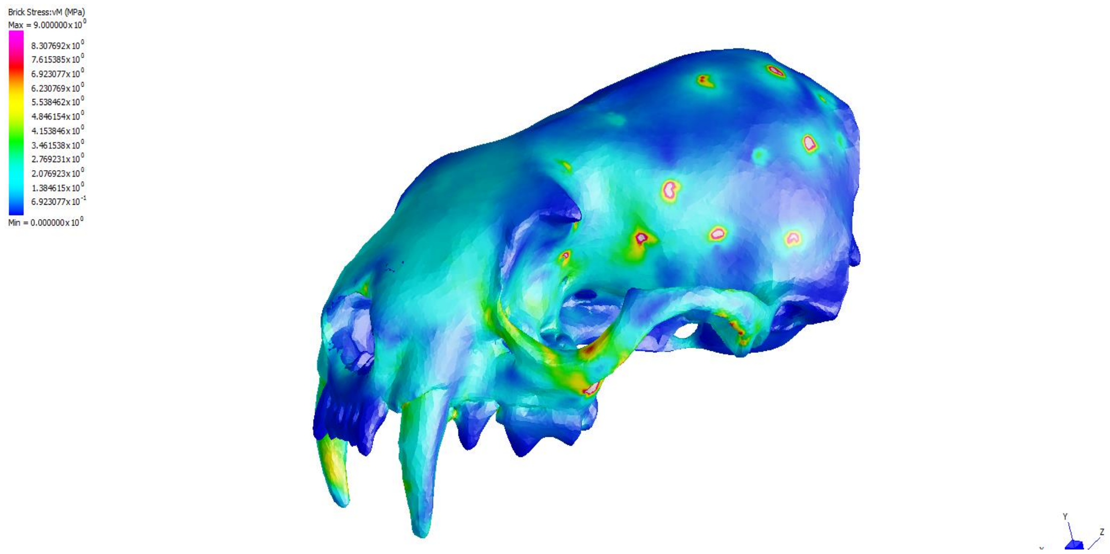

Supplementary Figure 12: Finite element model of a marbled polecat (*Vormela peregusna*) cranium. The models in Supplementary Figures 7 to 55 have been scaled to the same colour range. Models are not to scale.

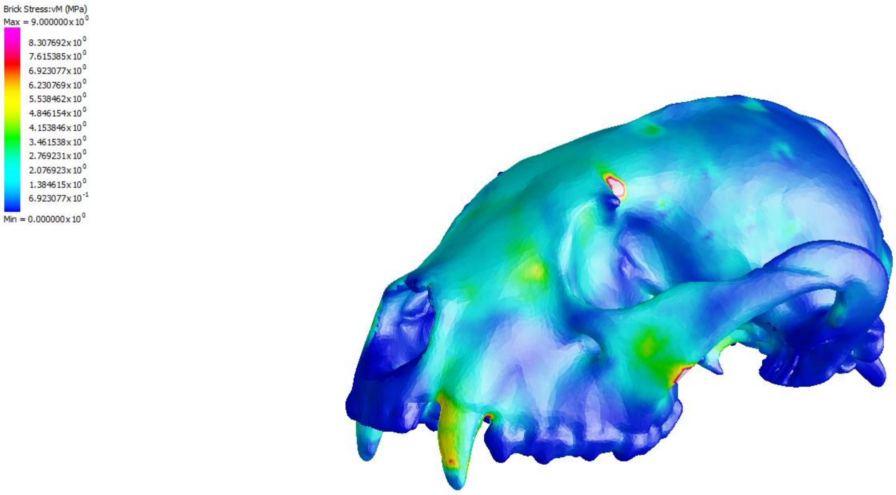

Supplementary Figure 13: Finite element model of a red panda (*Ailurus fulgens*) cranium. The models in Supplementary Figures 7 to 55 have been scaled to the same colour range. Models are not to scale.

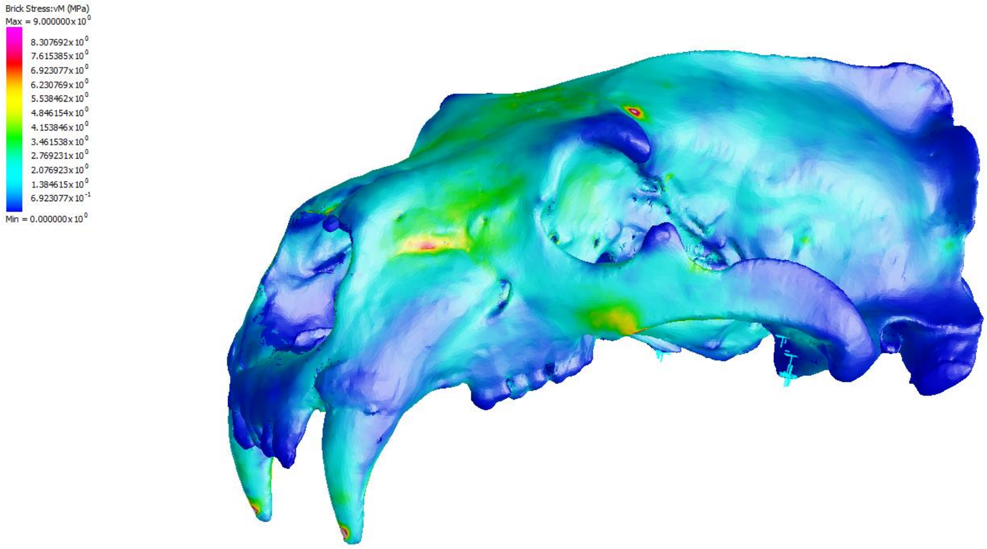

Supplementary Figure 14: Finite element model of a polar bear (*Ursus maritimus*) cranium. The models in Supplementary Figures 7 to 55 have been scaled to the same colour range. Models are not to scale.

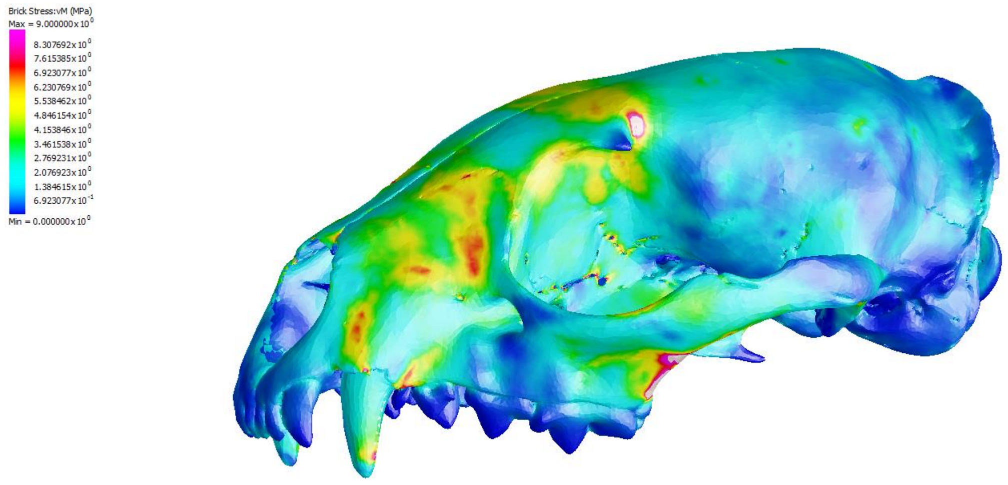

Supplementary Figure 15: Finite element model of a fossa (*Cryptoprocta ferox*) cranium. The models in Supplementary Figures 7 to 55 have been scaled to the same colour range. Models are not to scale.

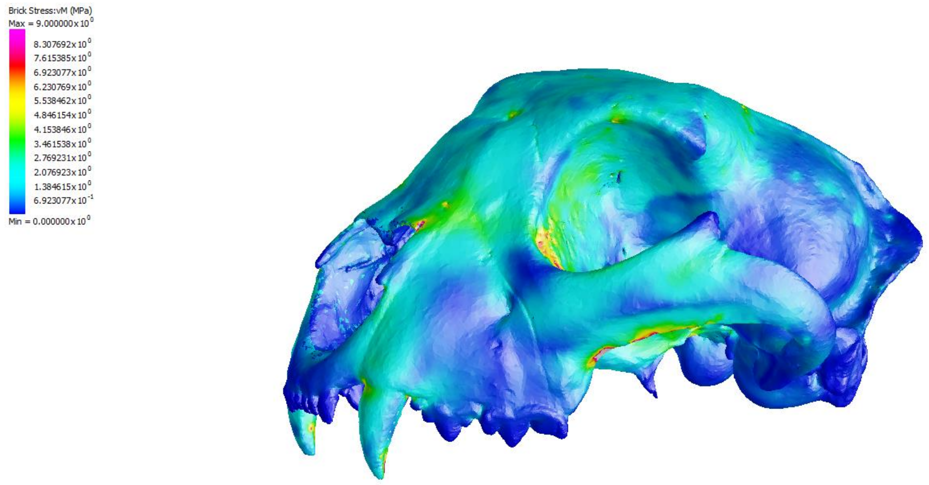

Supplementary Figure 16: Finite element model of a Cheeta (*Acinonyx jubatus*) cranium. The models in Supplementary Figures 7 to 55 have been scaled to the same colour range. Models are not to scale.

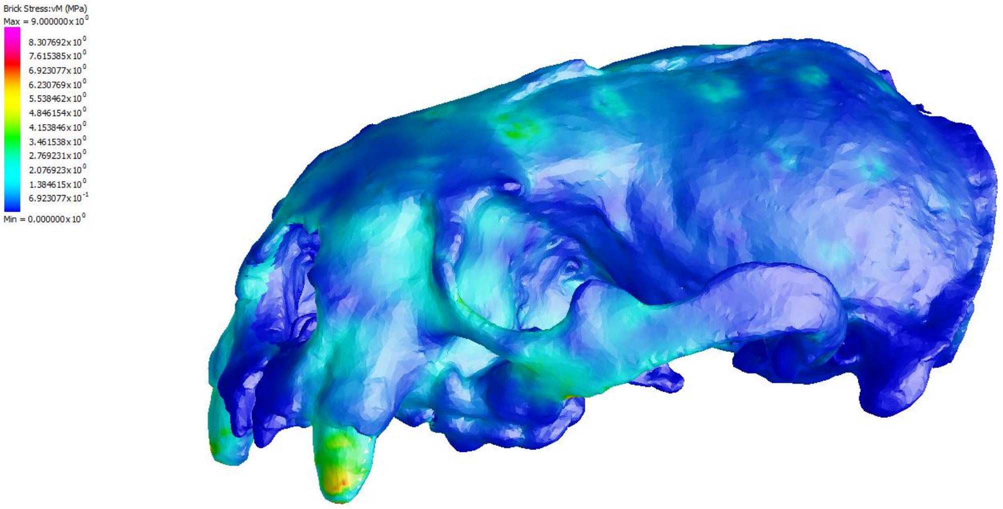

Supplementary Figure 7: Finite element model of an African clawless otter (*Aonyx capensis*) cranium. The models in Supplementary Figures 7 to 55 have been scaled to the same colour range. Models are not to scale.

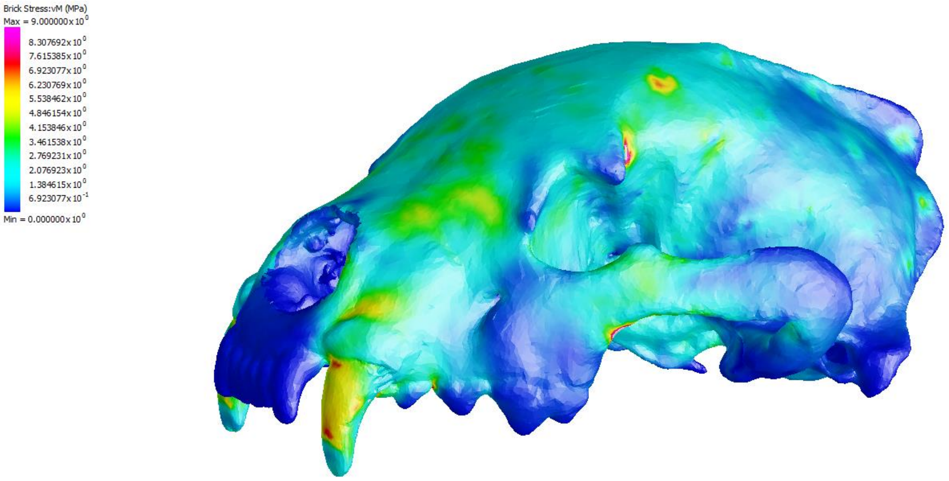

Supplementary Figure 18: Finite element model of a Wolverine (*Gulo gulo*) cranium. The models in Supplementary Figures 7 to 55 have been scaled to the same colour range. Models are not to scale.

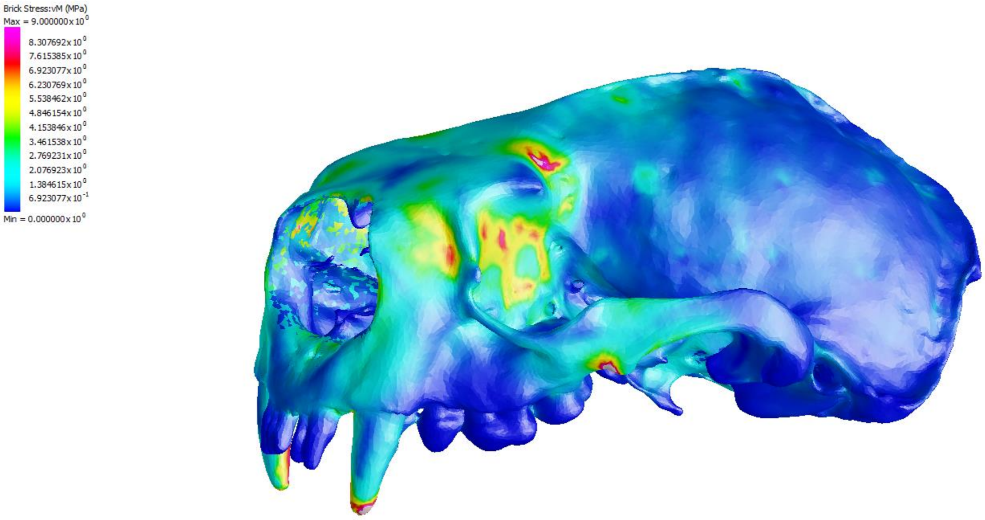

Supplementary Figure 19: Finite element model of a sea otter (*Enhydra lutris*) cranium. The models in Supplementary Figures 7 to 55 have been scaled to the same colour range. Models are not to scale.

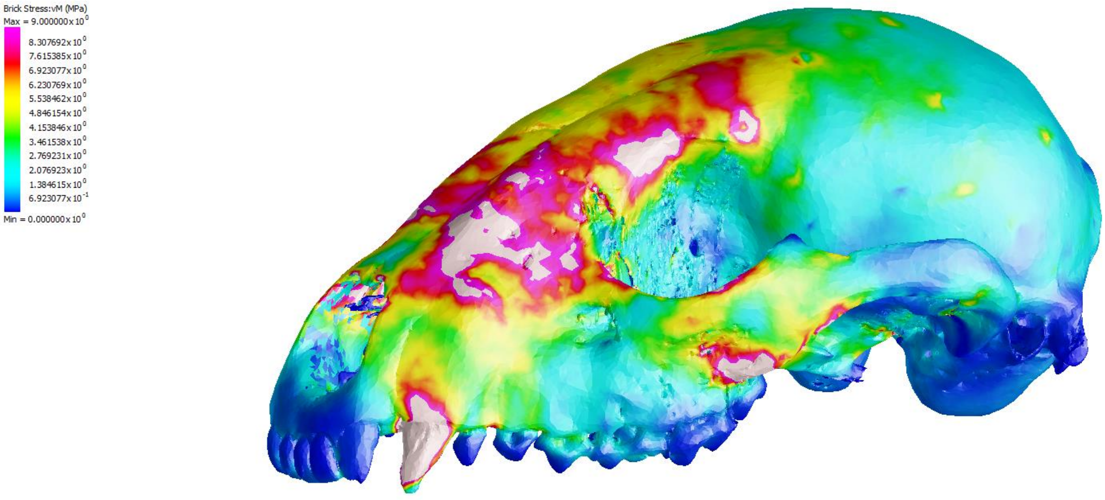

Supplementary Figure 20: Finite element model of a raccoon (*Procyon lotor*) cranium. The models in Supplementary Figures 7 to 55 have been scaled to the same colour range. Models are not to scale.

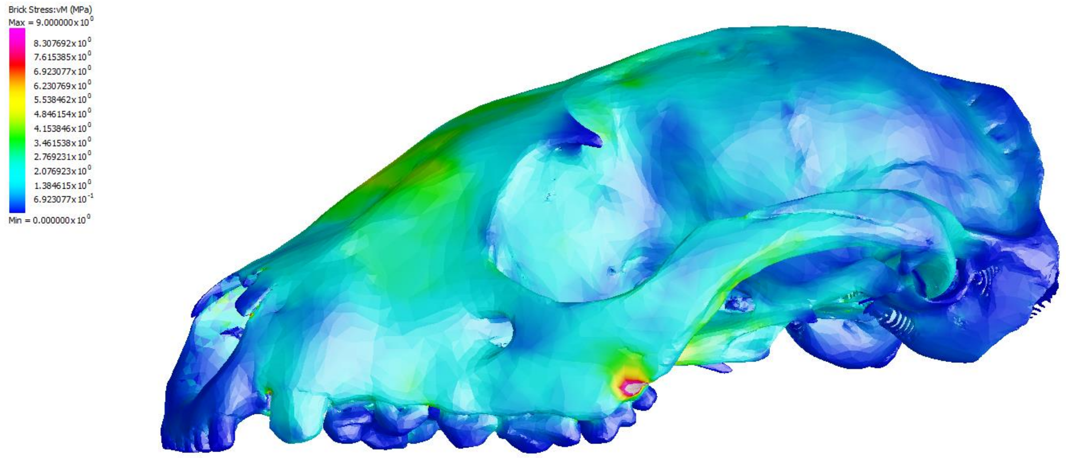

Supplementary Figure 21: Finite element model of a palm civet (*Paradoxurus hermaphroditus*) cranium. The models in Supplementary Figures 7 to 55 have been scaled to the same colour range. Models are not to scale.

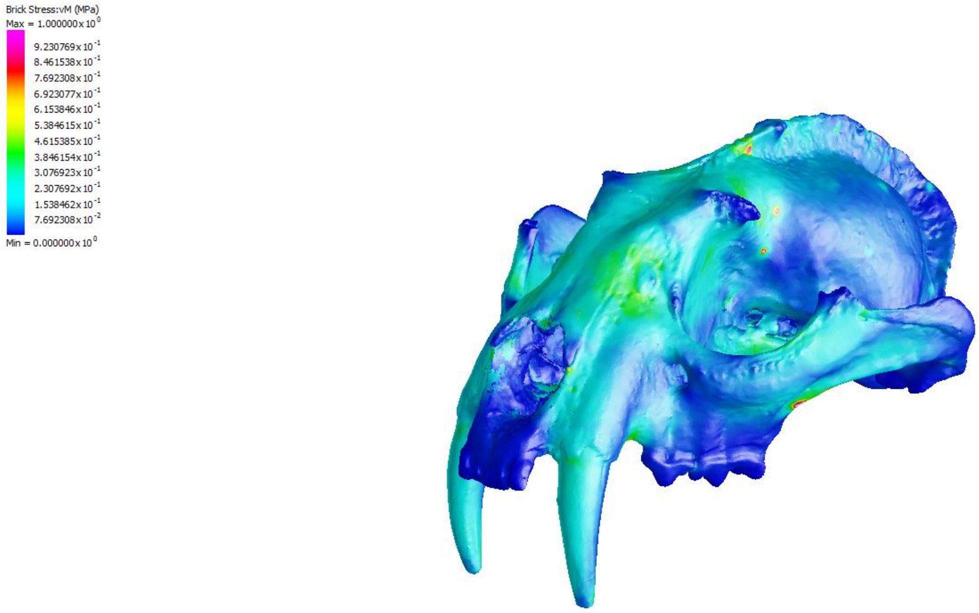

Supplementary Figure 22: Finite element model of a clouded leopard (*Neofelis nebulosa*) cranium. The models in Supplementary Figures 7 to 55 have been scaled to the same colour range. Models are not to scale.

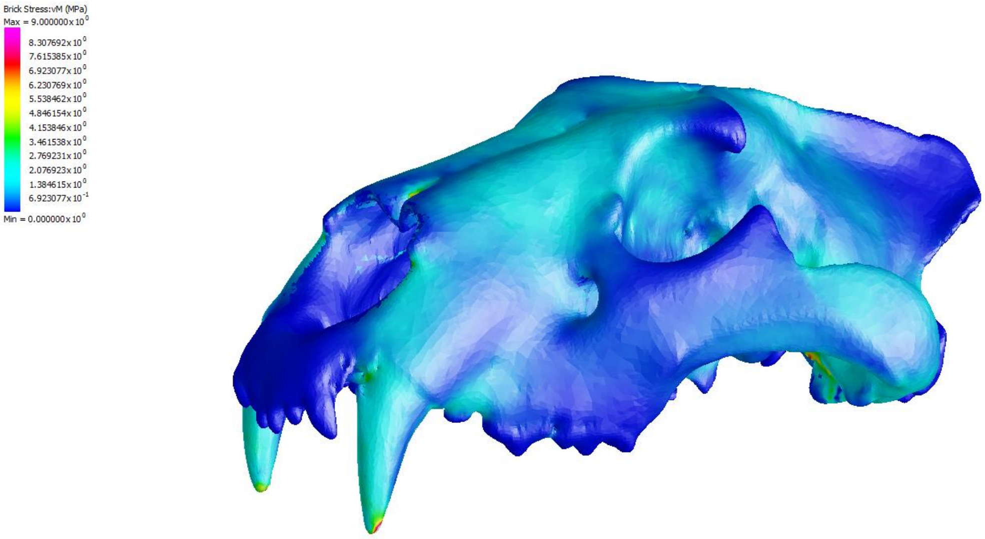

Supplementary Figure 23: Finite element model of a Lion (*Panthera leo*) cranium. The models in Supplementary Figures 7 to 55 have been scaled to the same colour range. Models are not to scale.

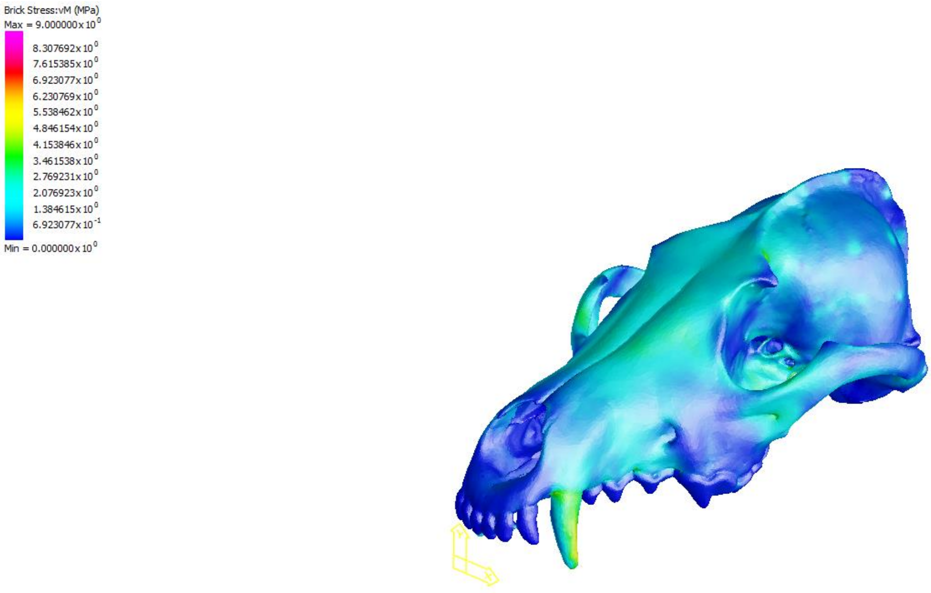

Supplementary Figure 24: Finite element model of a coyote (*Canis latrans*) cranium. The models in Supplementary Figures 7 to 55 have been scaled to the same colour range. Models are not to scale.

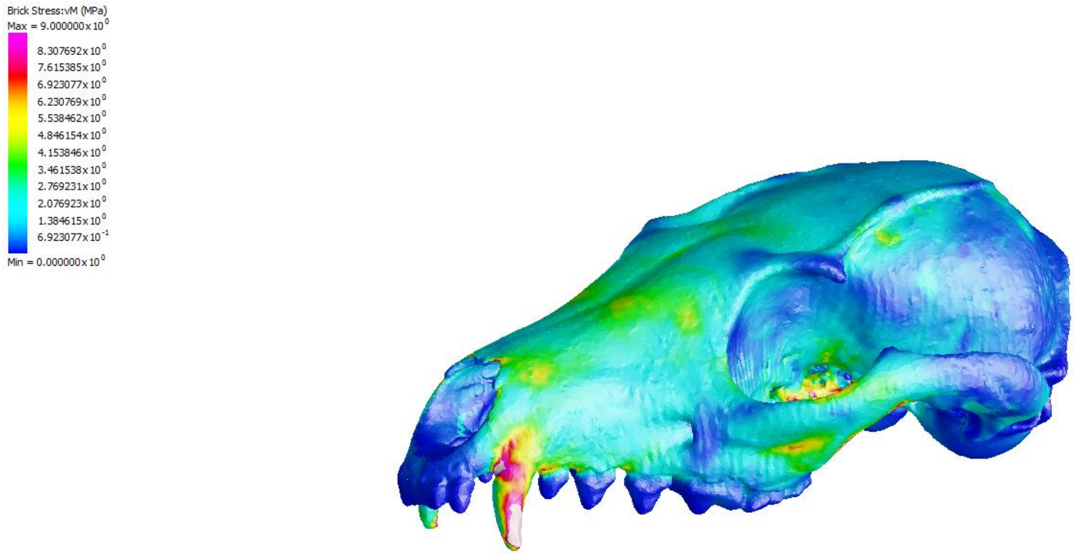

Supplementary Figure 25: Finite element model of a gray fox (*Urocyon cinereoargenteus*) cranium. The models in Supplementary Figures 7 to 55 have been scaled to the same colour range. Models are not to scale.

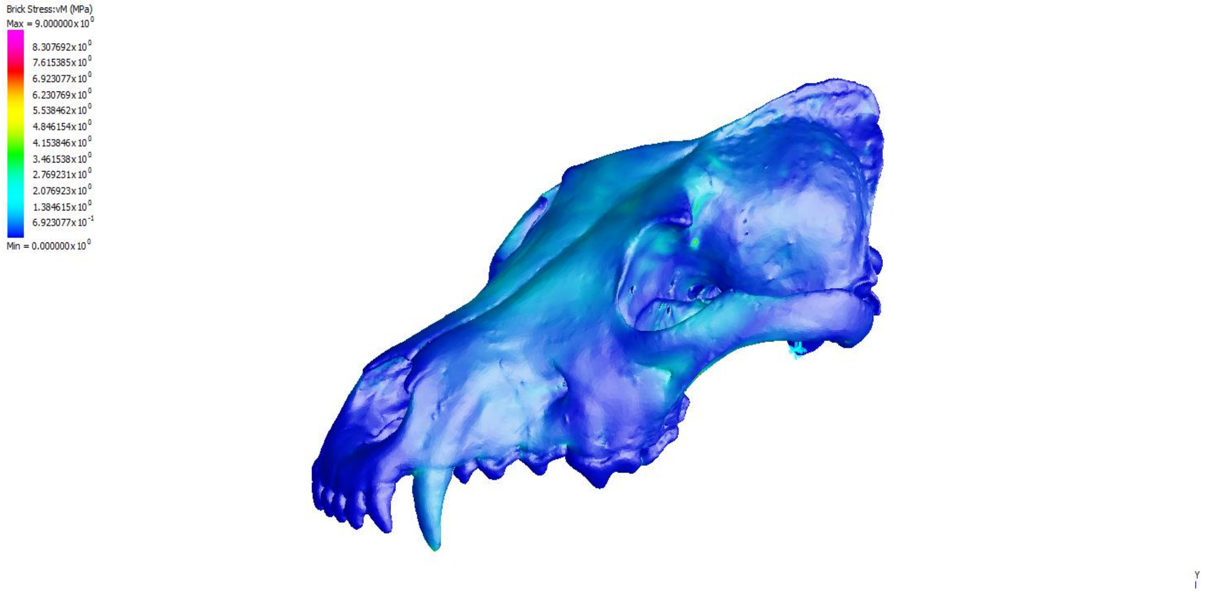

Supplementary Figure 26: Finite element model of a grey wolf (*Canis lupus*) cranium. The models in Supplementary Figures 7 to 55 have been scaled to the same colour range. Models are not to scale.

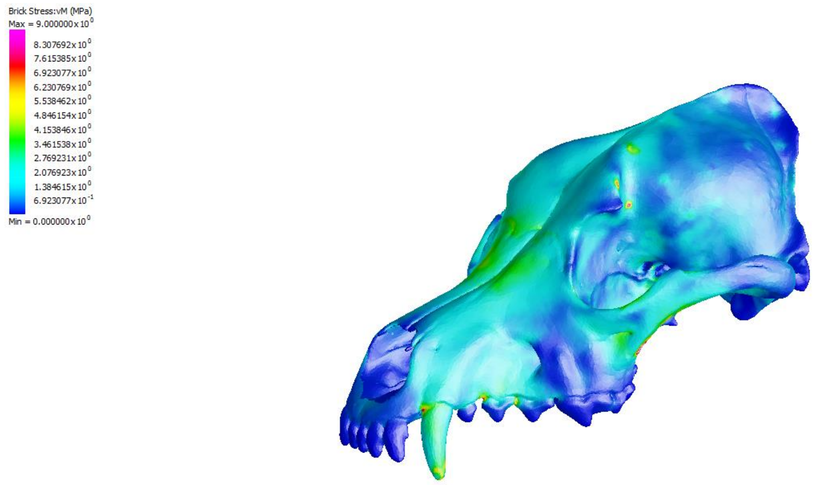

Supplementary Figure 27: Finite element model of a singing dog (*Canis lupus hallstromi*) cranium. The models in Supplementary Figures 7 to 55 have been scaled to the same colour range. Models are not to scale.

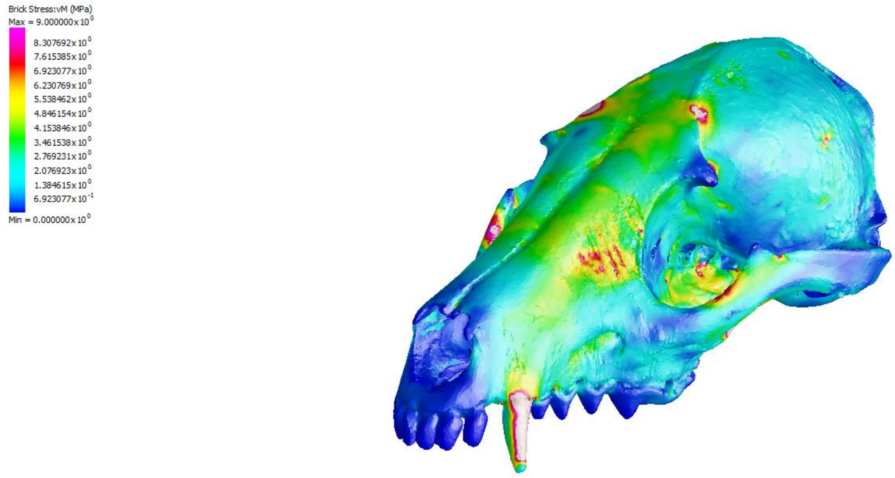

Supplementary Figure 28: Finite element model of a raccoon dog (*Nyctereutes procyonoides*) cranium. The models in Supplementary Figures 7 to 55 have been scaled to the same colour range. Models are not to scale.

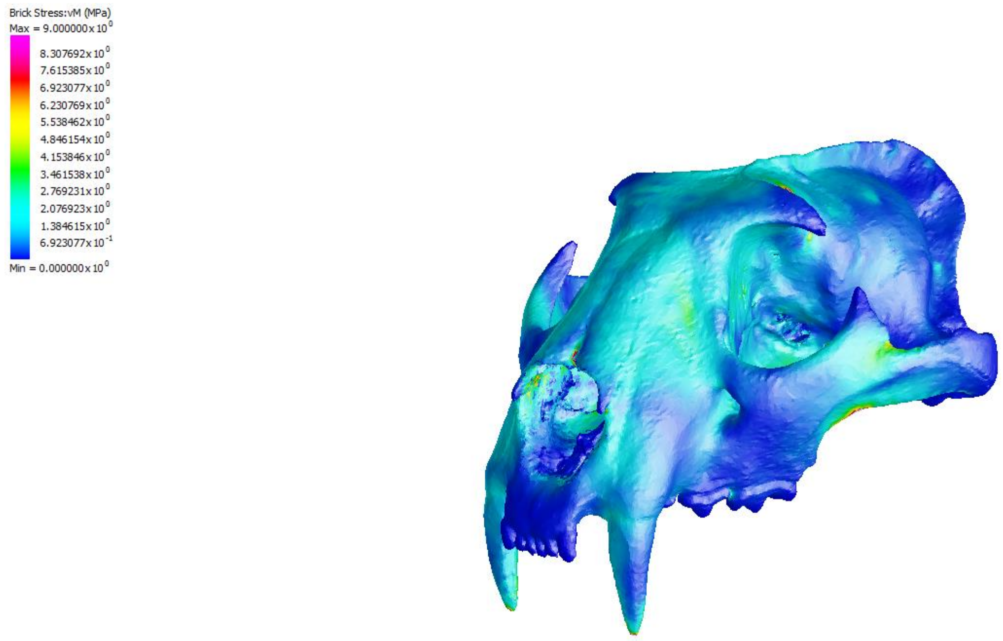

Supplementary Figure 29: Finite element model of a leopard (*Panthera pardus*) cranium. The models in Supplementary Figures 7 to 55 have been scaled to the same colour range. Models are not to scale.

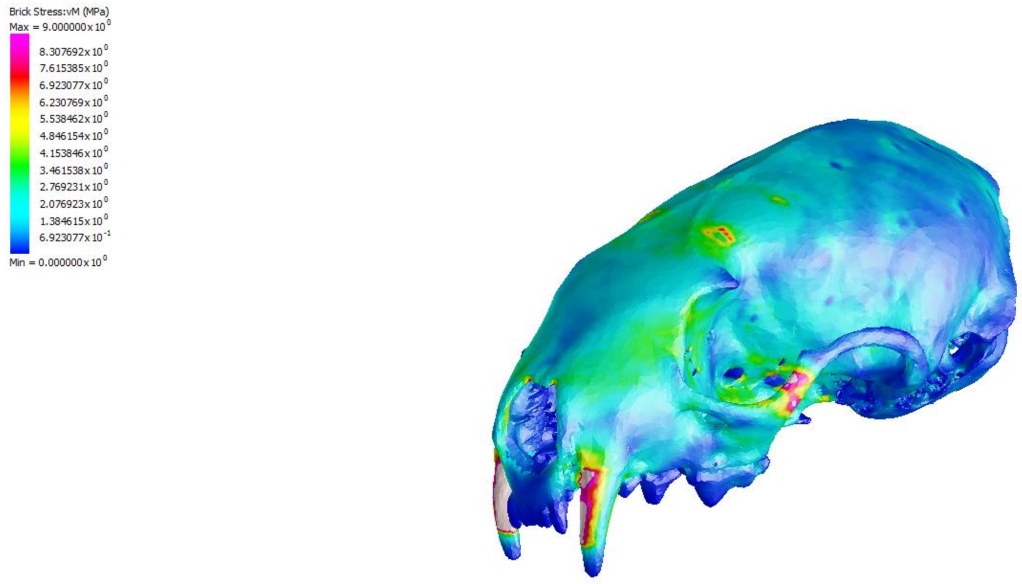

Supplementary Figure 30: Finite element model of a long tail weasel (*Neogale frenata*) cranium. The models in Supplementary Figures 7 to 55 have been scaled to the same colour range. Models are not to scale.

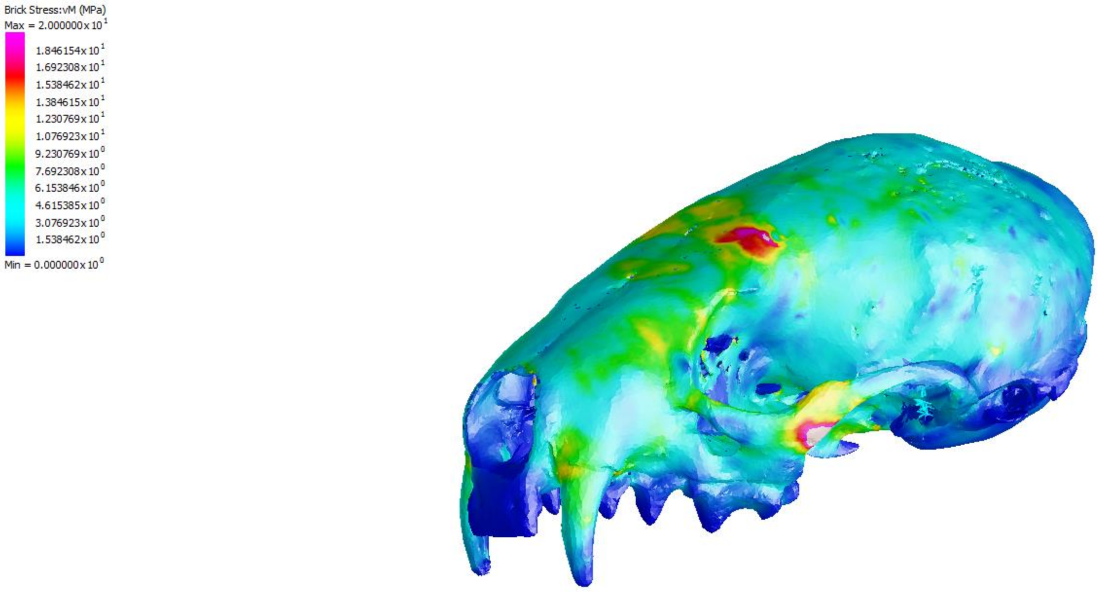

Supplementary Figure 31: Finite element model of a Mink (*Neogale vison*) cranium. The models in Supplementary Figures 7 to 55 have been scaled to the same colour range. Models are not to scale.

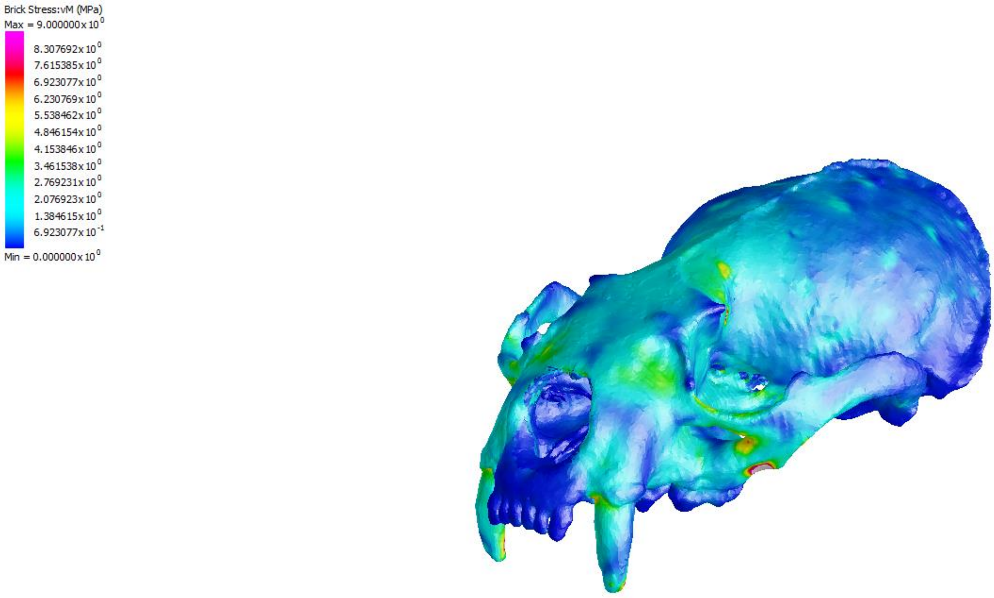

Supplementary Figure 32: Finite element model of a European otter (*Lutra lutra*) cranium. The models in Supplementary Figures 7 to 55 have been scaled to the same colour range. Models are not to scale.

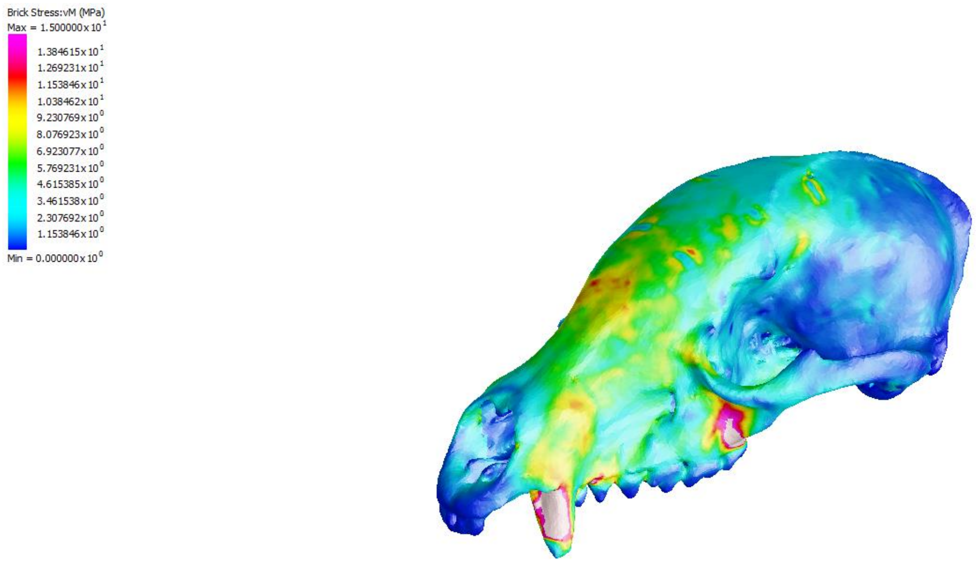

Supplementary Figure 33: Finite element model of a Southern American coati (*Nasua nasua*) cranium. The models in Supplementary Figures 7 to 55 have been scaled to the same colour range. Models are not to scale.

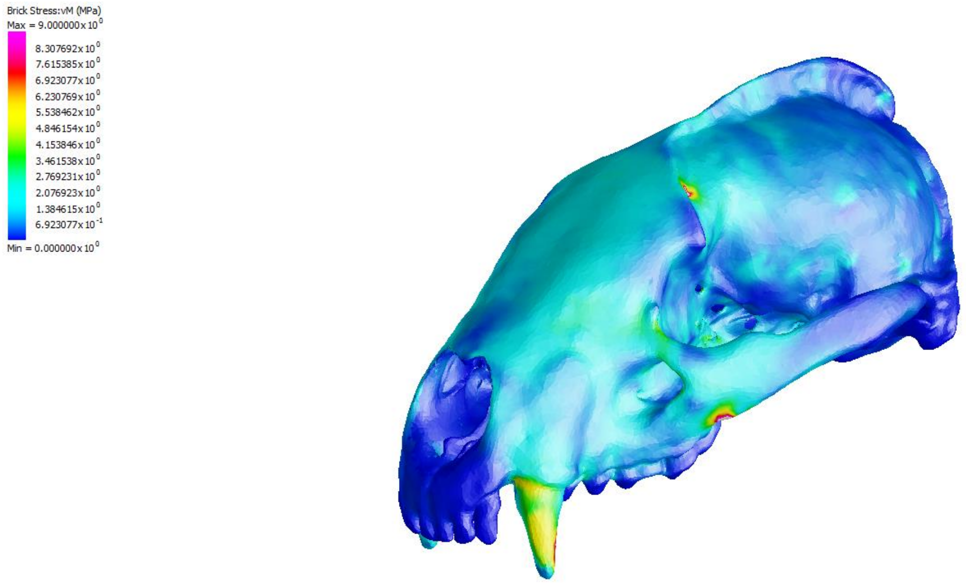

Supplementary Figure 34: Finite element model of a European badger (*Meles meles*) cranium. The models in Supplementary Figures 7 to 55 have been scaled to the same colour range. Models are not to scale.

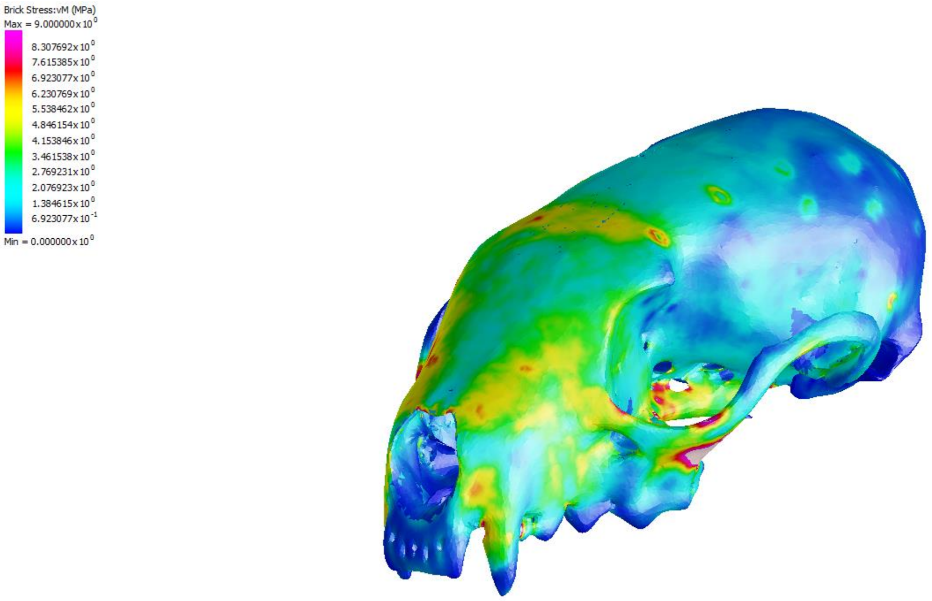

Supplementary Figure 35: Finite element model of a beech marten (*Martes foina*) cranium. The models in Supplementary Figures 7 to 55 have been scaled to the same colour range. Models are not to scale.

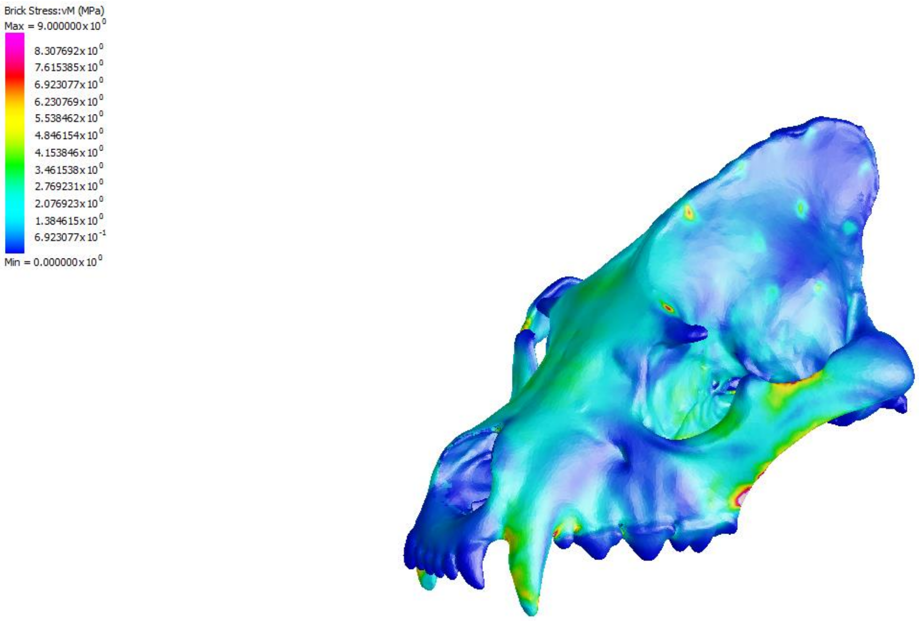

Supplementary Figure 36: Finite element model of a brown hyena (*Parahyaena brunnea*) cranium. The models in Supplementary Figures 7 to 55 have been scaled to the same colour range. Models are not to scale.

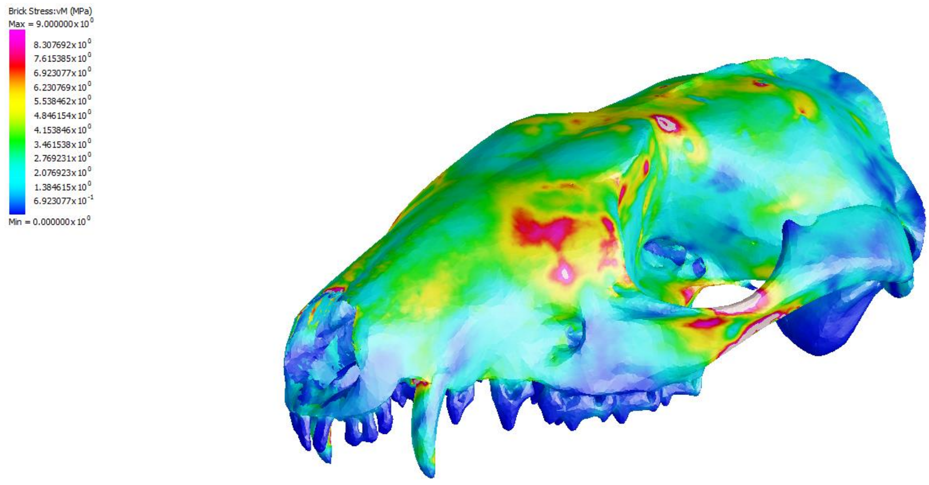

Supplementary Figure 37: Finite element model of a northern quoll (*Dasyurus hallucatus*) cranium. The models in Supplementary Figures 7 to 55 have been scaled to the same colour range. Models are not to scale.

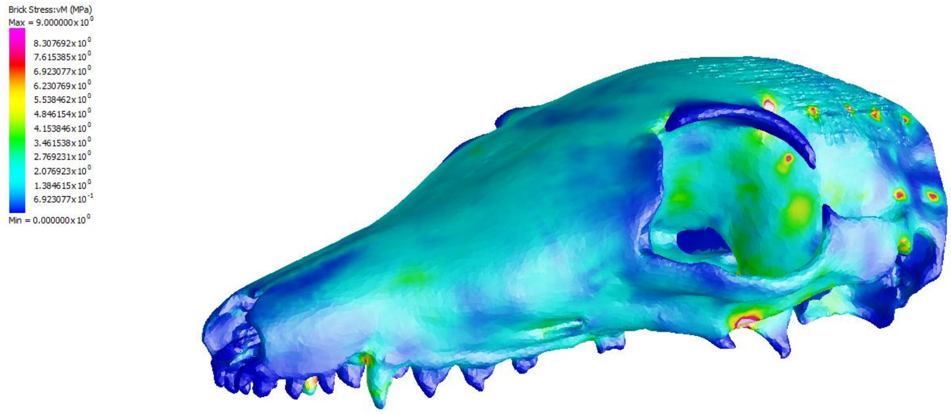

Supplementary Figure 38: Finite element model of a numbat (*Myrmecobius fasciatus*) cranium. The models in Supplementary Figures 7 to 55 have been scaled to the same colour range. Models are not to scale.

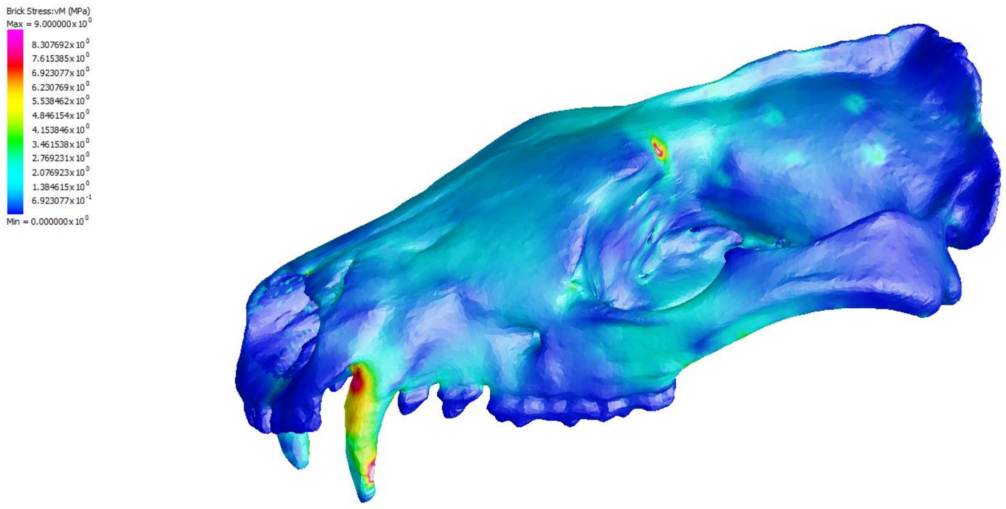

Supplementary Figure 39: Finite element model of a spotted-tailed quoll (*Dasyurus maculatus*) cranium. The models in Supplementary Figures 7 to 55 have been scaled to the same colour range. Models are not to scale.

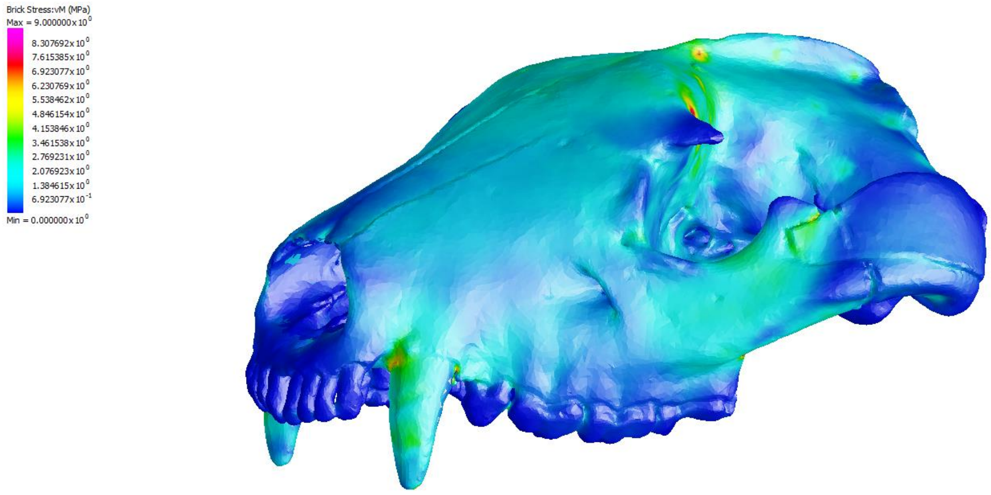

Supplementary Figure 40: Finite element model of a Tasmanian devil (*Sarcophilus harrisii*) cranium. The models in Supplementary Figures 7 to 55 have been scaled to the same colour range. Models are not to scale.

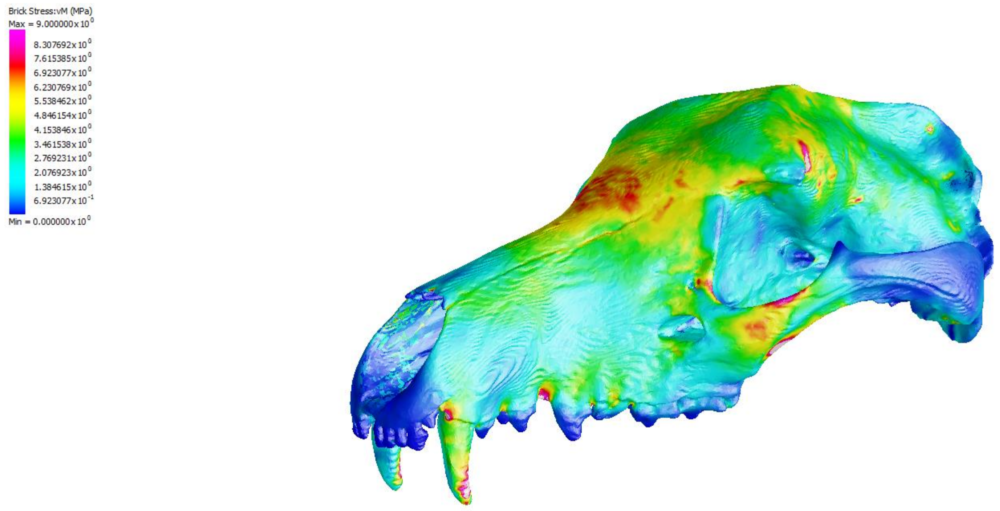

Supplementary Figure 41: Finite element model of a thylacine (*Thylacinus cynocephalus*) cranium. The models in Supplementary Figures 7 to 55 have been scaled to the same colour range. Models are not to scale.

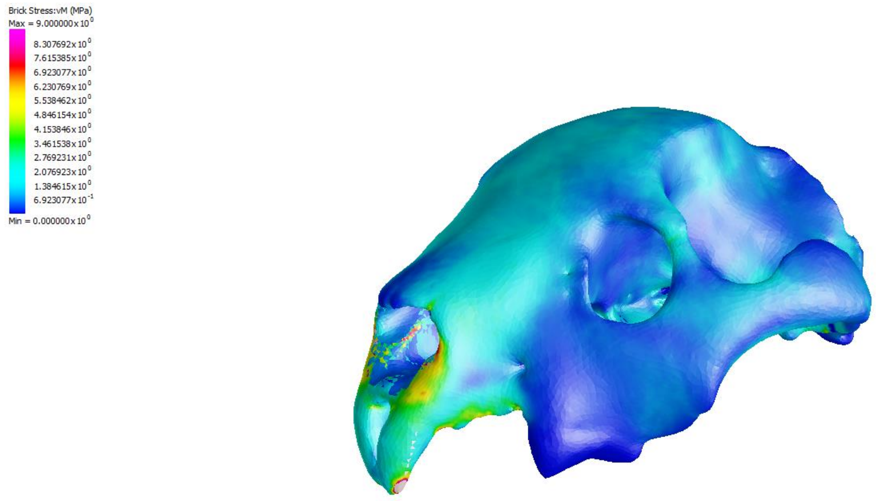

Supplementary Figure 42: Finite element model of a thylacoleo (*Thylacoleo carnifex*) cranium. The models in Supplementary Figures 7 to 55 have been scaled to the same colour range. Models are not to scale.

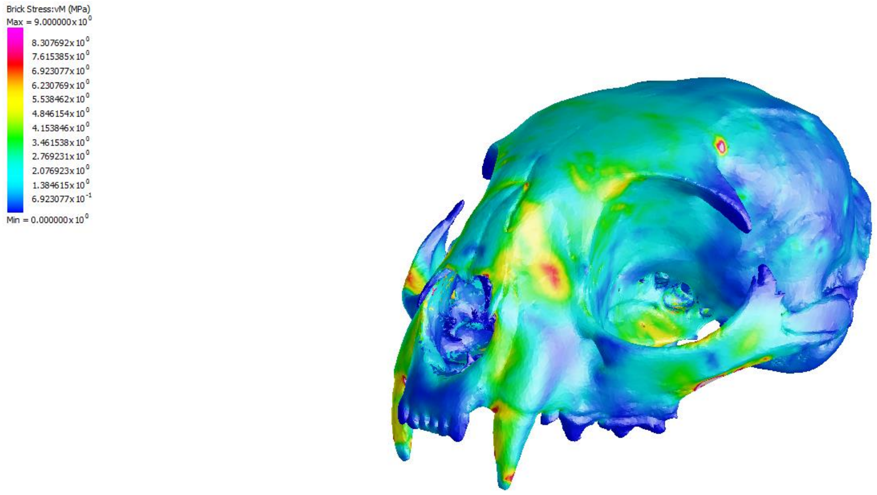

Supplementary Figure 44: Finite element model of an ocelot (*Leopardus pardalis*) cranium. The models in Supplementary Figures 7 to 55 have been scaled to the same colour range. Models are not to scale.

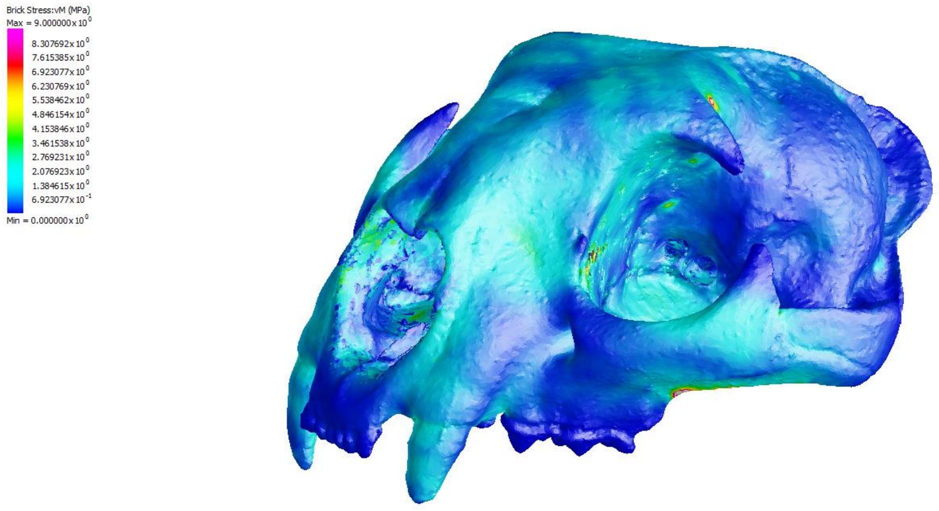

Supplementary Figure 44: Finite element model of a puma (*Puma concolor*) cranium. The models in Supplementary Figures 7 to 55 have been scaled to the same colour range. Models are not to scale.

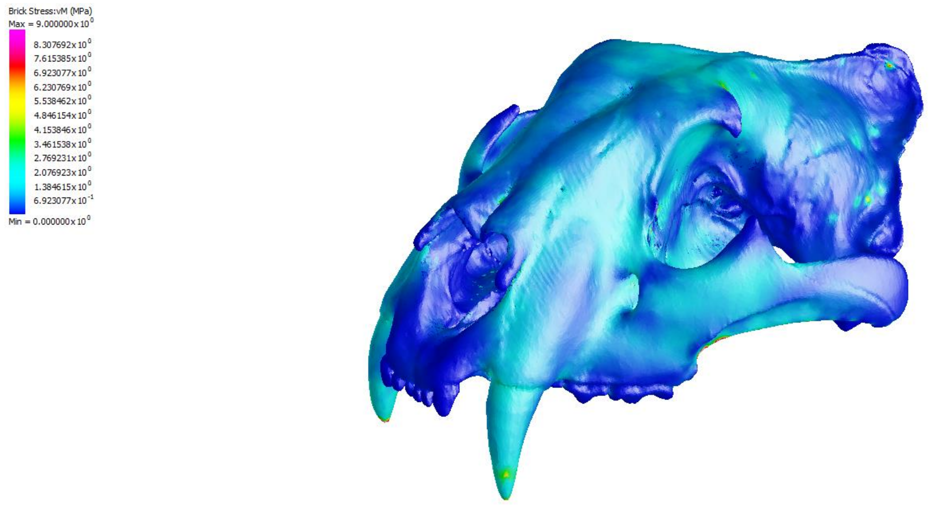

Supplementary Figure 45: Finite element model of a tiger (*Panthera tigris*) cranium. The models in Supplementary Figures 7 to 55 have been scaled to the same colour range. Models are not to scale.

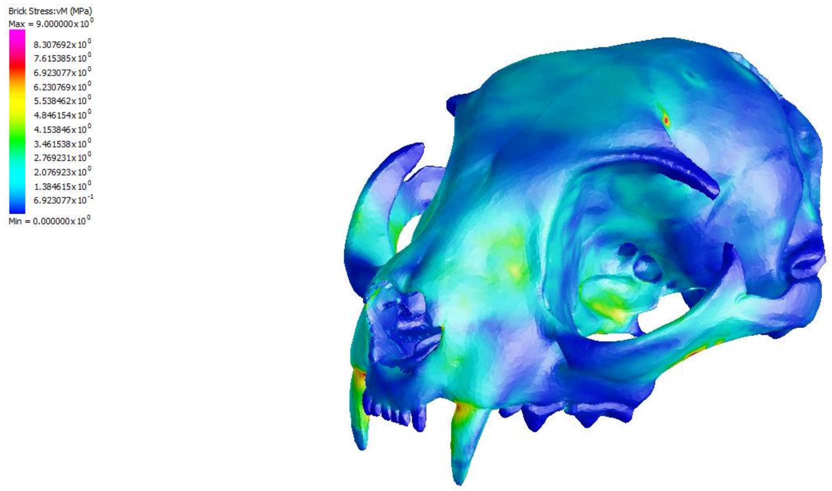

Supplementary Figure 46: Finite element model of a wildcat (*Felis silvestris*) cranium. The models in Supplementary Figures 7 to 55 have been scaled to the same colour range. Models are not to scale.

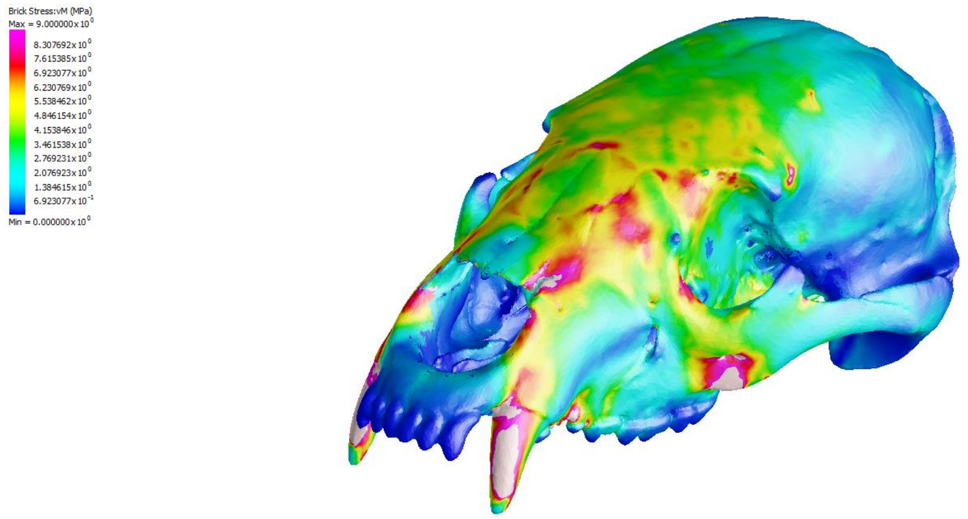

Supplementary Figure 47: Finite element model of an American black bear (*Ursus americanus*) cranium. The models in Supplementary Figures 7 to 55 have been scaled to the same colour range. Models are not to scale.

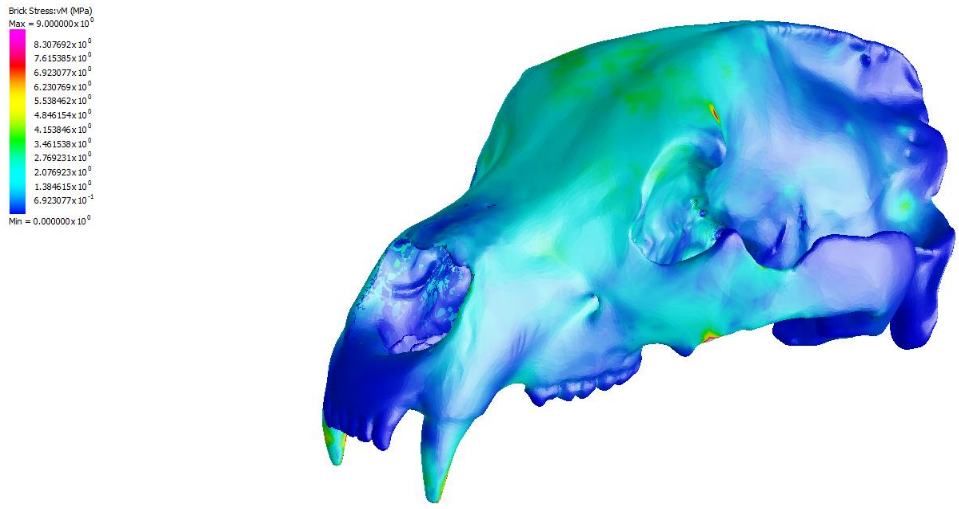

Supplementary Figure 48: Finite element model of a brown bear (*Ursus arctos*) cranium. The models in Supplementary Figures 7 to 55 have been scaled to the same colour range. Models are not to scale.

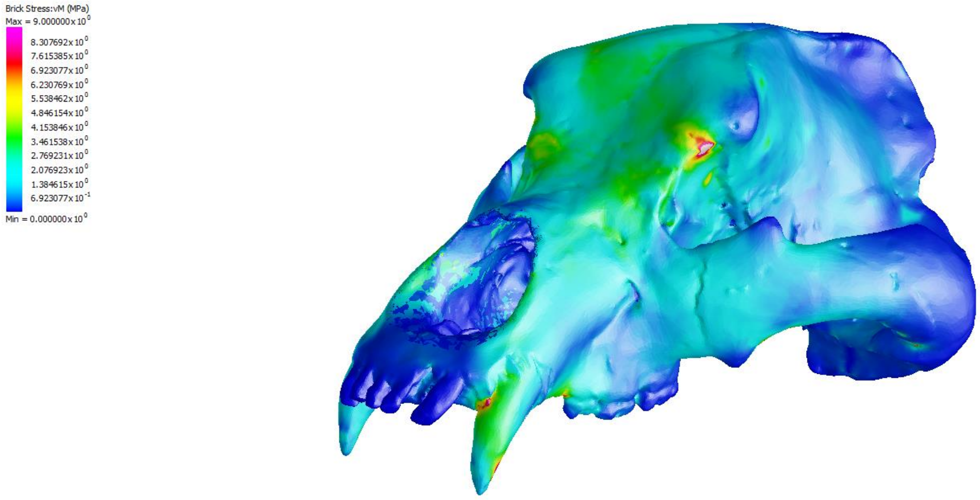

Supplementary Figure 49: Finite element model of a cave bear (*Ursus spelaeus*) cranium. The models in Supplementary Figures 7 to 55 have been scaled to the same colour range. Models are not to scale.

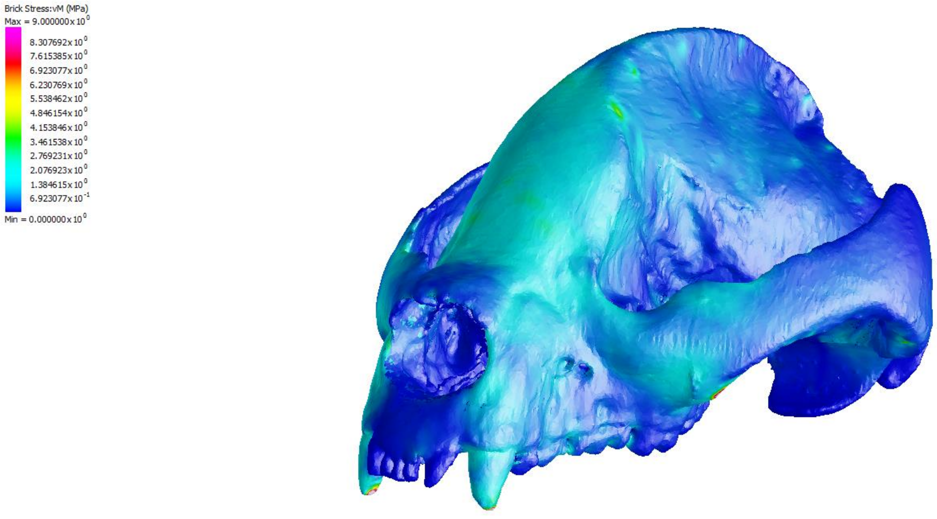

Supplementary Figure 50: Finite element model of a giant panda (*Ailuropoda melanoleuca*) cranium. The models in Supplementary Figures 7 to 55 have been scaled to the same colour range. Models are not to scale.

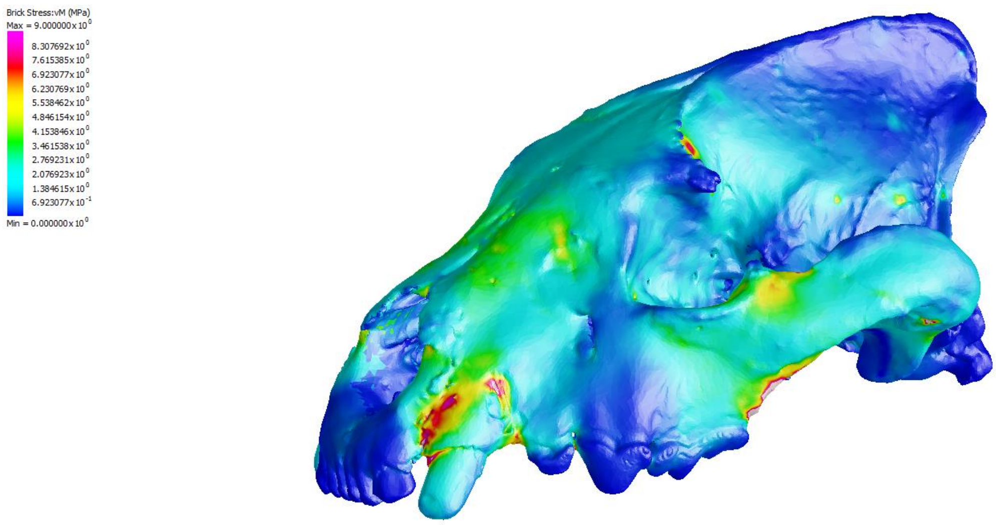

Supplementary Figure 51: Finite element model of a spotted hyena (*Crocuta crocuta*) cranium. The models in Supplementary Figures 7 to 55 have been scaled to the same colour range. Models are not to scale.

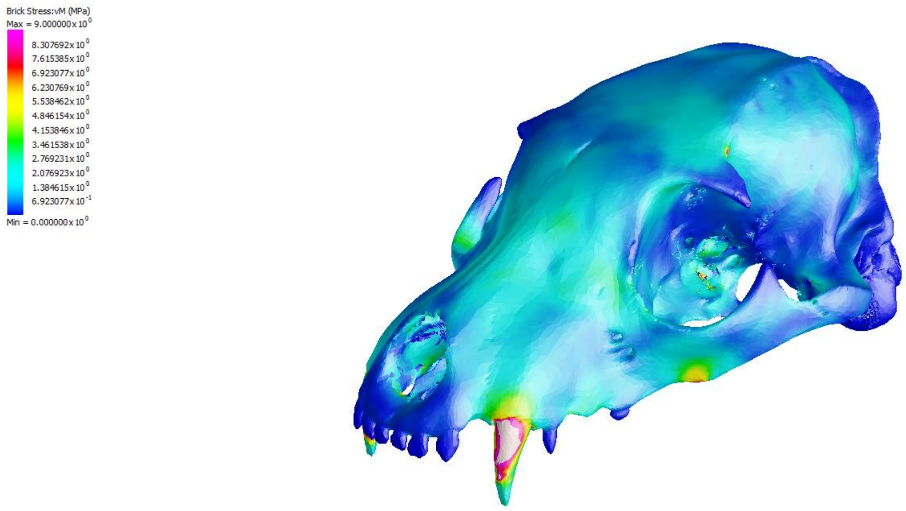

Supplementary Figure 52: Finite element model of an aardwolf (*Proteles cristata*) cranium. The models in Supplementary Figures 7 to 55 have been scaled to the same colour range. Models are not to scale.

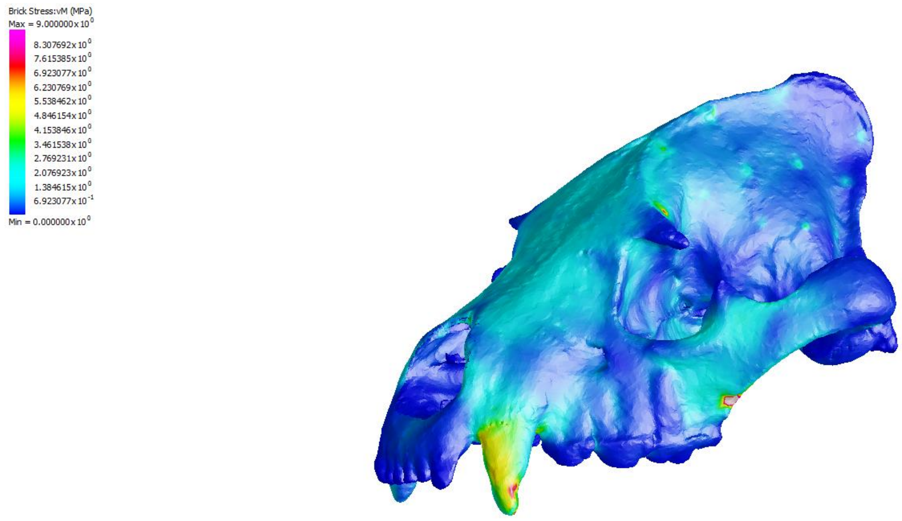

Supplementary Figure 53: Finite element model of a striped hyena (*Hyaena hyaena*) cranium. The models in Supplementary Figures 7 to 55 have been scaled to the same colour range. Models are not to scale.

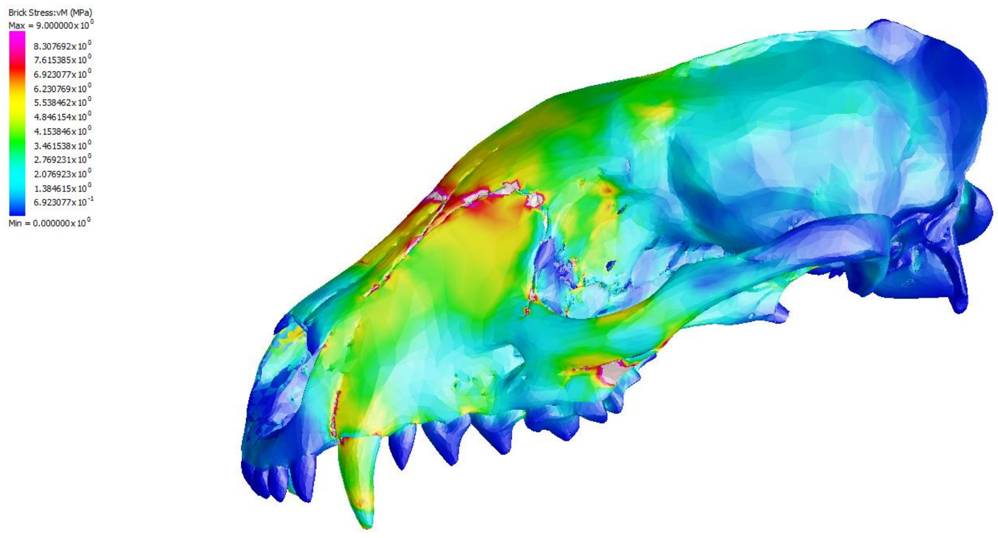

Supplementary Figure 54: Finite element model of an African civet (*Civettictis civetta*) cranium. The models in Supplementary Figures 7 to 55 have been scaled to the same colour range. Models are not to scale.

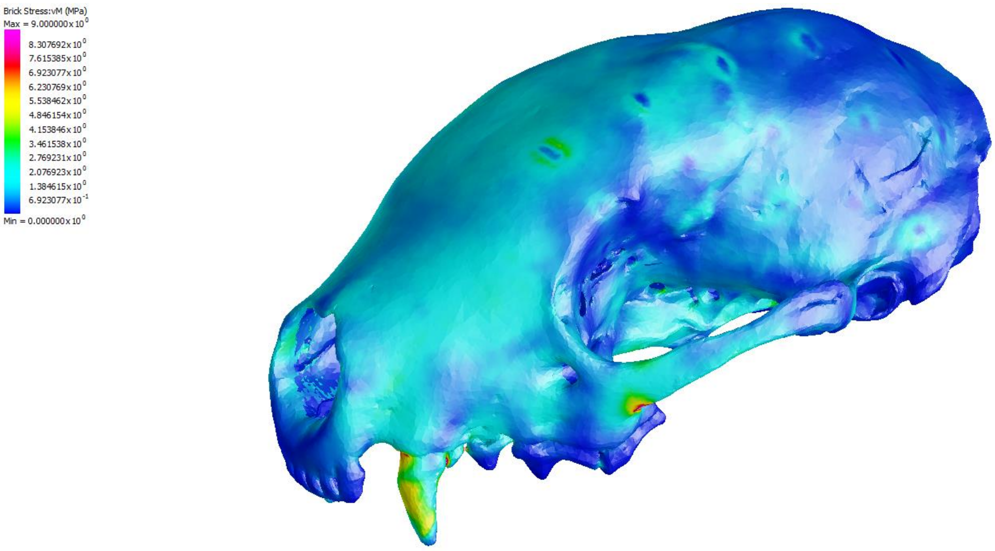

Supplementary Figure 55: Finite element model of a hooded skunk (*Mephitis macroura*) cranium. The models in Supplementary Figures 7 to 55 have been scaled to the same colour range. Models are not to scale.

References

1. Law, C. J. *et al.* Decoupled evolution of the cranium and mandible in carnivoran mammals. *Evolution (N. Y).* **76**, 2959–2974 (2022).
2. Dryden, I. L. & Mardia, K. V. *Statistical shape analysis: with applications in R*. vol. 995 (John Wiley & Sons, 2016).
3. Fruciano, C. Measurement error in geometric morphometrics. *Dev. Genes Evol.* **226**, 139–158 (2016).
4. Attard, M. R. G. *et al.* Virtual reconstruction and prey zize preference in the mid Cenozoic thylacinid, *Nimbacinus dicksoni* (Thylacinidae, Marsupialia). *PLoS One* **9**, e93088 (2014).
5. Sakamoto, M., Ruta, M. & Venditti, C. Extreme and rapid bursts of functional adaptations shape bite force in amniotes. *Proc. R. Soc. B Biol. Sci.* **286**, 20181932 (2019).
6. Hartstone-Rose, A., Hertzig, I. & Dickinson, E. Bite force and masticatory muscle architecture adaptations in the dietarily diverse Musteloidea (Carnivora). *Anat. Rec.* **302**,

2287–2299 (2019).

7. Christiansen and Adolfssen P, S. Bite forces, canine strength and skull allometry in carnivores (Mammalia, Carnivora). *J. Zool. London* **266**, 133–151 (2005).

8. McHenry, C. R., Wroe, S., Clausen, P. D., Moreno, K. & Cunningham, E. Supermodeled sabercat, predatory behavior in Smilodon fatalis revealed by high-resolution 3D computer simulation. *Proc. Natl. Acad. Sci. USA* **104**, 16010–16015 (2007).

9. Jordi, M.-N. *et al.* Computational and experimental approach of the phenomenology in the impact of a hen eggshell. in *International Conference on Computational Mechanics. 'World Academy of Science, Engineering and Technology'* 403–406 (2013).

10. Thomason, J. J. Cranial strength in relation to estimated biting forces in some mammals. *Can. J. Zool.* **69**, 2326–2333 (1991).

11. Penrose, F., Cox, P., Kemp, G. & Jeffery, N. Functional morphology of the jaw adductor muscles in the Canidae. *Anat. Rec.* **303**, 2878–2903 (2020).

12. Dickinson, E. *et al.* Evaluating bony predictors of bite force across the order Carnivora. *J. Morphol.* **282**, 1499–1513 (2021).

13. Chatterjee, S. A new coefficient of correlation. *J. Am. Stat. Assoc.* **116**, 2009–2022 (2021).

14. Felsenstein, J. Phylogenies and the comparative method. *Am. Nat.* **125**, 1–15 (1985).
