# Supplementary material for: Unexpectedly uneven distribution of functional trade-offs explains cranial morphological diversity in carnivores
Source: Nat Commun. 2024 Apr 16;15:3275. doi: 10.1038/s41467-024-47620-x (PMC11021405; doi:10.1038/s41467-024-47620-x)
Supplement: Supplementary file 3 — Description of Additional Supplementary Files [file 41467_2024_47620_MOESM3_ESM.pdf]

Description of Additional Supplementary Materials.

Supplementary Data 1. List of specimens used for the morphological analyses and related accession codes.

Supplementary Data 2. List of the specimens used in the biomechanical analyses and related accession codes.

Supplementary Data 3. Table presenting bite force estimates generated in this study compared with bite force estimates generated from previous publications including the same species.
