# Supplementary material for: Unexpectedly uneven distribution of functional trade-offs explains cranial morphological diversity in carnivores
Source: Nat Commun. 2024 Apr 16;15:3275. doi: 10.1038/s41467-024-47620-x (PMC11021405; doi:10.1038/s41467-024-47620-x)
Supplement: Supplementary file 7 — Reporting Summary [file 41467_2024_47620_MOESM7_ESM.pdf]

nature portfolio

Corresponding author(s): Gabriele Sansalone
Last updated by author(s): Mar 27, 2024

Reporting Summary

Nature Portfolio wishes to improve the reproducibility of the work that we publish. This form provides structure for consistency and transparency in reporting. For further information on Nature Portfolio policies, see our Editorial Policies and the Editorial Policy Checklist.

Statistics

For all statistical analyses, confirm that the following items are present in the figure legend, table legend, main text, or Methods section.

n/a	Confirmed	
☐	☒	The exact sample size (*n*) for each experimental group/condition, given as a discrete number and unit of measurement
☐	☒	A statement on whether measurements were taken from distinct samples or whether the same sample was measured repeatedly
☐	☒	The statistical test(s) used AND whether they are one- or two-sided<br>*Only common tests should be described solely by name; describe more complex techniques in the Methods section.*
☐	☒	A description of all covariates tested
☐	☒	A description of any assumptions or corrections, such as tests of normality and adjustment for multiple comparisons
☐	☒	A full description of the statistical parameters including central tendency (e.g. means) or other basic estimates (e.g. regression coefficient) AND variation (e.g. standard deviation) or associated estimates of uncertainty (e.g. confidence intervals)
☐	☒	For null hypothesis testing, the test statistic (e.g. F, t, r) with confidence intervals, effect sizes, degrees of freedom and P value noted<br>*Give P values as exact values whenever suitable.*
☐	☒	For Bayesian analysis, information on the choice of priors and Markov chain Monte Carlo settings
☐	☒	For hierarchical and complex designs, identification of the appropriate level for tests and full reporting of outcomes
☐	☒	Estimates of effect sizes (e.g. Cohen's d, Pearson's r), indicating how they were calculated

Our web collection on statistics for biologists contains articles on many of the points above.

Software and code

Policy information about availability of computer code

Data collection	Materialise Mimics v21; Geomagic Studio 2014; IDAV Landmark Editor; Strand7 V3.0
Data analysis	R statistical software v4.3.1; Morpho v2.12; RRphylo v2.8.0; BayesTraits v.4.0.

For manuscripts utilizing custom algorithms or software that are central to the research but not yet described in published literature, software must be made available to editors and reviewers. We strongly encourage code deposition in a community repository (e.g. GitHub). See the Nature Portfolio guidelines for submitting code & software for further information.

Data

Policy information about availability of data

All manuscripts must include a data availability statement. This statement should provide the following information, where applicable:

- Accession codes, unique identifiers, or web links for publicly available datasets
- A description of any restrictions on data availability
- For clinical datasets or third party data, please ensure that the statement adheres to our policy

All data, including biomechanical simulations, required to replicate this study are available at https://doi.org/10.6084/m9.figshare.23553648. Shape data performance data are available in the DR_shape.rda and in the DR_w.rda file. Source data are provided within the codes included in the file Code_Rscript.r. Within the script there are instructions to load separate .rda file that allow to reproduce Figures 1 to 6 and Supplementary Figures 1,4-6. Specimens accession codes are available in Supplementary Data 1 and 2.

Research involving human participants, their data, or biological material

Policy information about studies with human participants or human data. See also policy information about sex, gender (identity/presentation), and sexual orientation and race, ethnicity and racism.

Reporting on sex and gender	NA
Reporting on race, ethnicity, or other socially relevant groupings	NA
Population characteristics	NA
Recruitment	NA
Ethics oversight	NA

Note that full information on the approval of the study protocol must also be provided in the manuscript.

Field-specific reporting

Please select the one below that is the best fit for your research. If you are not sure, read the appropriate sections before making your selection.

☒ Life sciences ☐ Behavioural & social sciences ☐ Ecological, evolutionary & environmental sciences

For a reference copy of the document with all sections, see nature.com/documents/nr-reporting-summary-flat.pdf

Life sciences study design

All studies must disclose on these points even when the disclosure is negative.

Sample size	Sample size was based to cover the extant placentals and marsupials variability. Sample size fo fossil species was chosen based on availability, conservation status, and to ensure the broadest phylogenetic sampling.
Data exclusions	No data were excluded.
Replication	Semi-automatic landmarking procedure has been assessed using different templates returning consistent results. Different algorithms to build the adaptive landscape have been tested and the best one have been chosen by means of RMSE. We replicated our analysis by employing two distinct metrics as proxy of force: von Mises stress measured from the FEA models and Bite force estimated directly from the FEA simulations. We performed our Bayesian analysis on a distribution of trees to account for sampling, phylogenetic uncertainty in tree topology and branch lengths. Furthermore, we measured evolutionary rates using RRphylo function overfitRR repeated 100 times.
Randomization	Sample was organised into groups based on taxonomic assignment.
Blinding	Blinding was not possible as all analyses required a priori knowledge of fossil identity in order to accurately reconstruct the skull morphology and biomechanical properties.

Reporting for specific materials, systems and methods

We require information from authors about some types of materials, experimental systems and methods used in many studies. Here, indicate whether each material, system or method listed is relevant to your study. If you are not sure if a list item applies to your research, read the appropriate section before selecting a response.

Materials & experimental systems

n/a	Involved in the study	
☒	☐	Antibodies
☒	☐	Eukaryotic cell lines
☐	☒	Palaeontology and archaeology
☒	☐	Animals and other organisms
☒	☐	Clinical data
☒	☐	Dual use research of concern
☒	☐	Plants

Methods

n/a	Involved in the study	
☒	☐	ChIP-seq
☒	☐	Flow cytometry
☒	☐	MRI-based neuroimaging

Palaeontology and Archaeology

Specimen provenance	Specimens included in the biomechanical analyses are stored in different Australian institutions as detailed in Supplementary Data 1 and 2. These specimens were accessed by GC during 2014, which obtained authorization to access from the curators of the different collections.
Specimen deposition	All fossils are accessioned. Museums, online repositories and specimen numbers are provided in Supplementary Data 1 and 2.
Dating methods	No new dates are provided. First and last occurrence dates for all species were taken from literature and provided in Supplementary Information.

☒ Tick this box to confirm that the raw and calibrated dates are available in the paper or in Supplementary Information.

Ethics oversight	No ethical approval or guidance was required for this study.

Note that full information on the approval of the study protocol must also be provided in the manuscript.

Plants

Seed stocks	No plants were used in this study
Novel plant genotypes	NA
Authentication	NA
